# Correlation analysis of aroma, rhizosphere microbiota, and active constituents in Qianhu from different origins

**DOI:** 10.3389/fmicb.2026.1881890

**Published:** 2026-10-07

**Authors:** Yue Dong, Bin Deng, Zihao Gu, Yiping Qin, Qi Huang, Han Luo, Dongmei Xie, Congbin Liu

**Affiliations:** 1 School of Pharmacy, Anhui University of Chinese Medicine, Hefei, China; 2 Institute of TCM Resources Protection and Development, Anhui Academy of Chinese Medicine, Hefei, China

**Keywords:** Qianhu, geo-authenticity, GC-IMS, rhizosphere microorganisms, aroma components

## Abstract

**Background:**

This study aimed to investigate the differences and intrinsic relationships among aroma components, rhizosphere microorganisms, and active constituents of Qianhu from the geo-authentic production area of Ningguo, Anhui Province, and other production areas, including Guizhou and Chongqing.

**Methods:**

Gas chromatography–ion mobility spectrometry (GC-IMS) was used to analyze aroma components, and relative odor activity value (ROAV) analysis was applied to identify key aroma compounds and potential markers for origin discrimination. High-throughput sequencing was used to characterize bacterial and fungal communities in rhizosphere soils. High-performance liquid chromatography (HPLC) was performed to determine the contents of praeruptorin A (PA) and praeruptorin B (PB). Correlation analysis was further conducted to clarify the relationships among aroma components, rhizosphere microorganisms, and active constituents.

**Results:**

A total of 69 volatile components were identified. Among them, 10 key aroma compounds with ROAV values greater than 1 showed significantly higher ROAV values in Ningguo samples than in samples from other regions, indicating a pronounced aroma advantage. The dominant bacterial taxa in the rhizosphere of the geo-authentic production area were *norank_o__Vicinamibacterales* and *norank_o__Terriglobales*, while the dominant fungal taxa were *Didymella* and *Neocosmospora*. Active constituent analysis showed that sample G2 from Guizhou had the highest PA content (1.76%), whereas sample ND4 from Ningguo had the highest PB content (0.25%). Correlation analysis revealed that within the bacterial community, *Gaiellales* showed positive correlation trends with multiple components and exhibited the broadest range of associations. Within the fungal community, *Neocosmospora* and *Bisifusarium* showed positive correlation trends with multiple aroma components, suggesting potential roles in flavor formation. Among bacteria, *norank_f__Vicinamibacteraceae* showed a positive correlation trend with PA, while *norank_o__Rokubacteriales* showed a positive correlation trend with PB. Among fungi, *Neocosmospora* showed a negative correlation trend with PA and a positive correlation trend with PB. Regarding aroma components, heptaldehyde-M showed a positive correlation trend with PA, while unidentified compound 6 showed a negative correlation trend with PA and a positive correlation trend with PB.

**Conclusion:**

Qianhu from the geo-authentic region (Ningguo) showed certain advantageous trends in aroma intensity and key microbial taxa. The accumulation of PA and PB may be associated with distinct microbial functional groups. These findings provide preliminary exploratory insights into the microecological mechanisms underlying the geo-authenticity formation of Qianhu.

## 1 Introduction

*Peucedanum praeruptorum* Dunn (Qianhu), a traditional Chinese medicinal herb derived from the dried roots of Apiaceae plants, is commonly used to relieve coughs, reduce phlegm, and disperse wind-heat (Chinese Pharmacopoeia Commission, 2025). Its major chemical constituents include coumarins, volatile oils, phenanthrenequinones, organic acids, and sterols (Wang et al., 2024). Modern pharmacological studies have demonstrated that Qianhu exhibits expectorant (Lee et al., 2017), antitussive (Ozaki et al., 2023), antiasthmatic (Xiong et al., 2012), anti-inflammatory (Zhang et al., 2010), and sedative effects (Deng et al., 2022), as well as potential activities in anti-tumor therapy (Hu et al., 2023) and cardiovascular disease prevention (Sarkhail et al., 2013). Among these, volatile oils are considered one of the key bioactive components contributing to its clinical efficacy (Chen et al., 2021).

Geographical investigations indicate that the production areas of Qianhu have remained relatively consistent over time, with major traditional regions centered in Zhejiang, Anhui, and Jiangxi provinces, and emerging regions in Sichuan, Guizhou, and Chongqing (Liu, T. L. et al., 2022). Ningguo City in Anhui Province is recognized as a geo-authentic production area, where the product, known as “Ning Qianhu,” is characterized by large size, dark skin, elongated roots, a yellow interior, and a strong aroma (Li and Zhang, 2014; Yang et al., 2012).

Previous studies using high-performance liquid chromatography (HPLC) fingerprinting have classified Qianhu samples from multiple regions into distinct groups, including geo-authentic areas such as Ningguo and Bozhou in Anhui, other regions such as Hunan, Guizhou, and Chongqing, and high-altitude areas in Yunnan (Xie et al., 2024). In addition to coumarins, Qianhu contains abundant volatile compounds. For the analysis of these volatile components, GC–MS has been employed in previous studies to identify a wide range of volatile compounds in Qianhu, including alkanes, esters, ketones, sesquiterpenes, aromatics, and naphthoquinones (Song et al., 2022). Among them, α-pinene, sabinol, aromadendrene, terpinolene, α-farnesene, and longifolene account for over 60% of the total volatile peak area and are considered the major contributors to its characteristic aroma (Yu et al., 2007).

GC-IMS offers improved resolution over traditional IMS (Yang et al., 2014) and provides advantages including high sensitivity, rapid analysis, and minimal sample preparation compared with techniques such as electronic nose (Mu et al., 2024), GC-olfactometry (Li et al., 2020), and GC–MS (Tao and Yan, 2022). However, it should be noted that GC-IMS cannot resolve structural isomers as effectively as GC–MS, and its spectral library is less comprehensive than NIST GC–MS databases. Since this study focuses on comparing volatile profile differences among Qianhu samples from different origins rather than detailed structural identification, GC-IMS serves as an efficient tool for volatile fingerprinting. It has been widely applied in the identification of medicinal material origins (Chen et al., 2022; Guo et al., 2021; Li et al., 2021; Liu et al., 2023), species differentiation (An et al., 2024; Wang, R. et al., 2021), and analysis of volatile compounds at different developmental stages (Feng et al., 2021; Ma et al., 2019). In this study, GC-IMS, combined with principal component analysis (PCA) and ROAV, was employed to characterize and screen key aroma compounds in Qianhu from different origins, aiming to identify potential aroma quality markers of Ning Qianhu.

Rhizosphere microorganisms are essential components of soil ecosystems and play crucial roles in improving soil physicochemical properties (Philippot et al., 2013), enhancing nutrient uptake by plant roots (Xu et al., 2025), and suppressing soil-borne diseases (Ai et al., 2025), thereby contributing to the formation of geo-authentic medicinal materials (Cardone et al., 2019). They also regulate plant growth (Yu et al., 2024; Zahoor et al., 2022; Zhang et al., 2021), stress resistance (Dao et al., 2023; Ren et al., 2023; Ye et al., 2023), disease resistance (Han et al., 2021; Lu et al., 2021; Wang, Q. et al., 2021), and the biosynthesis of secondary metabolites (Korenblum et al., 2022; Lv et al., 2024; Wang et al., 2022). Previous studies have shown that inoculation with the fungus *Didymella* segeticola can significantly increase the yield of coumarins in *P. praeruptorum* (Liu, L. et al., 2022), suggesting that specific rhizosphere microorganisms may influence the accumulation of bioactive compounds in this medicinal plant. However, the mechanisms underlying this effect remain to be elucidated. Nonetheless, this observation points to a possible role of rhizosphere microbes in regulating secondary metabolism, warranting a broader investigation of the rhizosphere microbiome. Accordingly, the present study conducts a comprehensive survey of the rhizosphere microbial communities associated with Qianhu, aiming to provide foundational data and generate hypotheses for future research on plant–microbe interactions governing quality formation.

Traditional microbial culture methods detect only a small fraction of cultivable microorganisms and fail to reflect the true diversity and structure of microbial communities. In contrast, next-generation sequencing (NGS) technologies offer high throughput, low cost, and high accuracy, enabling comprehensive analysis of complex microbial communities, including low-abundance and previously unknown species (He, 2023; Tang et al., 2024). For the rhizosphere soil of *P. praeruptorum*, which may harbor unique microbial taxa associated with its geo-authentic quality formation, NGS-based approaches are particularly valuable for uncovering the full spectrum of microbial diversity and identifying candidate taxa that may contribute to the accumulation of bioactive compounds.

To date, no study has integrated the rhizosphere microbial composition, aroma components, and active constituents of Qianhu from geo-authentic and other production regions into a unified correlation analysis framework. Based on the above background, we propose the following working hypothesis: geographical origin shapes the composition of rhizosphere microbial communities, which in turn may influence the accumulation of volatile compounds and active constituents in Qianhu through microbe–plant interactions, such as nutrient mobilization, phytohormone modulation, and induction of plant defense/secondary metabolism pathways. This hypothesis is supported by previous studies showing that specific rhizosphere microorganisms can affect coumarin accumulation, and that plant secondary metabolism is often modulated by microbial partners. The present correlation-based study serves as an essential first step to identify candidate microbial taxa and metabolite markers for future mechanistic and inoculation-based validation studies.

In this study, GC-IMS was used to analyze aroma components in Qianhu from different regions, and ROAV was applied to identify key odor-active compounds. High-throughput sequencing was employed to characterize the diversity and dominant taxa of rhizosphere microbial communities. Additionally, HPLC was used to quantify PA and PB. Spearman correlation analysis was performed to evaluate the associations among key microbial taxa, aroma components, and major active components, providing a basis for identifying aroma quality markers and improving the cultivation of high-quality Qianhu.

## 2 Materials and methods

### 2.1 Instruments and reagents

A FlavourSpec® gas chromatography–ion mobility spectrometry system was purchased from G.A.S. (Dortmund, Germany). A YP10002B electronic balance was obtained from Shanghai Hengji Scientific Instrument Co., Ltd. A −80 °C freezer was purchased from Qingdao Haier Special Electric Appliance Co., Ltd. The E.Z.N.A.® Soil DNA Kit was obtained from Omega Bio-tek (USA), and FastPfu Polymerase was purchased from TransGen Biotech Co., Ltd. (Beijing, China). A T100 Thermal Cycler PCR system was obtained from BIO-RAD (USA), and a JY600C electrophoresis apparatus was purchased from Beijing Junyi Electrophoresis Equipment Co., Ltd. An MM400 mixer mill was obtained from Retsch (Germany), an LGJ-10 freeze dryer from Beijing Songyuan Freeze Dryer Technology Development Co., Ltd., and a D-78532 refrigerated centrifuge from Tuttlingen, Germany. High-throughput sequencing was performed using an Illumina NextSeq 2000 platform by Majorbio Bio-Pharm Technology Co., Ltd. (Shanghai, China). An Agilent 1,100 high-performance liquid chromatography system was purchased from Agilent Technologies (USA). AX324ZH and EX125DZH electronic balances were obtained from OHAUS Instruments Co., Ltd. (Changzhou, China), and an ME analytical balance was purchased from METTLER TOLEDO (Switzerland). A CM-RO-C2 ultrapure water system was obtained from Ningbo Dansbodon Environmental Protection Technology Co., Ltd., and an AS1510473 ultrasonic cleaner was purchased from Tianjin Autoscience Instrument Co., Ltd.

The n-ketones, including 2-butanone, 2-pentanone, 2-hexanone, 2-heptanone, 2-octanone, and 2-nonanone, were of analytical grade and purchased from Aladdin Co., Ltd. Nitrogen gas with a purity of 99.999% was used. The 20 mL headspace vials were obtained from Shandong Hanon Scientific Instrument Co., Ltd. An MXT-WAX capillary column (30 m × 0.53 mm, 1.0 μm) was purchased from Restek Corporation (USA). Reference standards of praeruptorin A (batch No. 73069-27-9, 20 mg) and praeruptorin B (batch No. 4970-26-7, 20 mg) were obtained from Chengdu Push Bio-technology Co., Ltd. Chromatographic-grade methanol was purchased from TEDIA Company Inc. (USA).

### 2.2 Experimental materials

A total of 20 batches of *Peucedanum praeruptorum* samples and corresponding rhizosphere soil samples were collected from different production areas. The plant materials were authenticated by Professor Xie Dongmei from Anhui University of Chinese Medicine as the dried roots of *Peucedanum praeruptorum* Dunn., and voucher specimens were deposited at the Herbarium of Anhui University of Chinese Medicine (specimen code: ACM-2024-001 to ACM-2024-020, with representative specimen No. ACM-2024-001). All sampling sites met the following strict inclusion criteria to minimize confounding factors: (1) identical seed source, all originating from the geo-authentic production region; (2) same cultivation company with consistent agronomic management practices; and (3) comparable cultivation duration and environmental conditions. The unbalanced sample sizes reflect the actual availability of sites that satisfied these criteria in each region.

Healthy, disease-free plants in good growth condition were selected using the five-point sampling method. Rhizosphere soil was collected using the shake-off method. Briefly, loosely adhering soil was removed by gentle shaking, and the soil that remained firmly attached to the root surface(operationally defined as rhizosphere soil, within approximately 2 mm of the root surface) was carefully collected using sterile brushes. Soil samples were placed in sterile sealed bags, transported to the laboratory on dry ice, and stored at −80 °C until further processing. Sample information is shown in Table 1.

**Table 1:** Sampling sites and sample information.

No.	Collection location	Collection time	Sample code
1	Sidu Town, Huichuan District, Zunyi City, Guizhou Province (106°54′10″E, 27°55′8″N)	Nov 2024	G11–G13
2	Shawan Town, Huichuan District, Zunyi City, Guizhou Province (106°52′52″E, 27°55′34″N)	Nov 2024	G21–G23
3	Shawan Town, Huichuan District, Zunyi City, Guizhou Province (106°52′46″E, 27°55′34″N)	Nov 2024	G31–G33
4	Hexi Town, Zheng’an County, Zunyi City, Guizhou Province (107°23′16″E, 28°27′49″N)	Nov 2024	G41–G43
5	Banzhu Town, Zheng’an County, Zunyi City, Guizhou Province (107°37′26″E, 28°35′2″N)	Nov 2024	G51–G53
6	Qiaozi Township, Pengshui Miao and Tujia Autonomous County, Chongqing (108°23′34″E, 29°23′51″N)	Dec 2024	C11–C13
7	Nanji Township, Ningguo City, Xuancheng City, Anhui Province (119°0′49″E, 30°24′21″N)	Dec 2024	NJ11–NJ13
8	Nanji Township, Ningguo City, Xuancheng City, Anhui Province (119°0′59″E, 30°24′17″N)	Dec 2024	NJ21–NJ23
9	Nanji Township, Ningguo City, Xuancheng City, Anhui Province (119°1′0″E, 30°24′12″N)	Dec 2024	NJ31–NJ33
10	Nanji Township, Ningguo City, Xuancheng City, Anhui Province (119°3′34″E, 30°23′19″N)	Dec 2024	NJ41–NJ43
11	Ningdun Town, Ningguo City, Xuancheng City, Anhui Province (119°6′40″E, 30°26′30″N)	Dec 2024	ND11–ND13
12	Ningdun Town, Ningguo City, Xuancheng City, Anhui Province (119°6′42″E, 30°26′1″N)	Dec 2024	ND21–ND23
13	Ningdun Town, Ningguo City, Xuancheng City, Anhui Province (119°6′46″E, 30°25′56″N)	Dec 2024	ND31–ND33
14	Ningdun Town, Ningguo City, Xuancheng City, Anhui Province (119°6′40″E, 30°26′22″N)	Dec 2024	ND41–ND43
15	Ningdun Town, Ningguo City, Xuancheng City, Anhui Province (119°6′7″E, 30°26′0″N)	Dec 2024	ND51–ND53
16	Zhongxi Town, Ningguo City, Xuancheng City, Anhui Province (119°11′4″E, 30°28′40″N)	Dec 2024	ZX11–ZX13
17	Zhongxi Town, Ningguo City, Xuancheng City, Anhui Province (119°11′23″E, 30°28′25″N)	Dec 2024	ZX21–ZX23
18	Zhongxi Town, Ningguo City, Xuancheng City, Anhui Province (119°12′56″E, 30°26′39″N)	Dec 2024	ZX31–ZX33
19	Zhongxi Town, Ningguo City, Xuancheng City, Anhui Province (119°12′52″E, 30°26′36″N)	Dec 2024	ZX41–ZX43
20	Zhongxi Town, Ningguo City, Xuancheng City, Anhui Province (119°13′57″E, 30°25′39″N)	Dec 2024	ZX51–ZX53

The sample codes follow this rule: letters denote the sampling region, the first digit denotes the sampling site within that region, and the last digit denotes the replicate. The abbreviations for the sampling regions and their corresponding sample codes are as follows: GZ corresponds to G11–G13, G21–G23, G31–G33, G41–G43, and G51–G53; CQ corresponds to C11–C13; NJ corresponds to NJ11–NJ13, NJ21–NJ23, NJ31–NJ33, and NJ41–NJ43; ND corresponds to ND11–ND13, ND21–ND23, ND31–ND33, ND41–ND43, and ND51–ND53; and ZX corresponds to ZX11–ZX13, ZX21–ZX23, ZX31–ZX33, ZX41–ZX43, and ZX51–ZX53.

### 2.3 Experimental methods

#### 2.3.1 GC-IMS

Fresh Qianhu samples were sliced, and 3.0 g of each sample was accurately weighed into a 20 mL headspace vial. Samples were incubated at 55 °C for 15 min before injection. Each sample was analyzed in triplicate.

The headspace injection conditions were as follows: incubation temperature, 55 °C; incubation time, 15 min; injection volume, 300 μL; splitless injection; incubation speed, 500 r/min; syringe temperature, 105 °C.

The GC conditions were as follows: column temperature, 60 °C; carrier gas, high-purity nitrogen (>99.999%); initial flow rate, 2.0 mL/min for 2 min, increased linearly to 10.0 mL/min within 8 min, then to 100.0 mL/min within 10 min, and maintained for 30 min. The total run time was 50 min, and the injection port temperature was 80 °C.

The IMS conditions were as follows: ionization source, tritium source; drift tube length, 53 mm; electric field strength, 500 V/cm; drift tube temperature, 45 °C; drift gas, high-purity nitrogen (>99.999%) at 75.0 mL/min; positive ion mode; gate opening time, 100 μs; gate voltage, 90 dgt.

#### 2.3.2 DNA extraction, PCR amplification, and high-throughput sequencing

Total genomic DNA of the microbial community was extracted using the E.Z.N.A.® Soil DNA Kit (Omega Bio-tek, Norcross, GA, USA) according to the manufacturer’s instructions. The quality of the extracted genomic DNA was assessed by 1% agarose gel electrophoresis, and DNA concentration and purity were determined using a NanoDrop 2000 spectrophotometer (Thermo Scientific, USA).

The bacterial 16S rRNA V5–V7 region and fungal ITS region were selected as target regions for PCR amplification and sequencing of rhizosphere bacteria and fungi of *P. praeruptorum*, respectively. The bacterial primers were 338F: 5′-ACTCCTACGGGAGGCAGCA-3′ and 806R: 5′-GGACTACHVGGGTWTCTAAT-3′ (Zeng and An, 2021). The fungal primers were ITS1F: 5′-CTTGGTCATTTAGAGGAAGTAA-3′ and ITS2R: 5′-GCTGCGTTCTTCATCGATGC-3′ (Gardes and Bruns, 1993). PCR products were recovered by 2% agarose gel electrophoresis and purified using a DNA gel extraction and purification kit. The purified products were quantified using Qubit 4.0. Sequencing libraries were prepared using the NEXTFLEX Rapid DNA-Seq Kit, and high-throughput sequencing was performed on an Illumina NextSeq 2000.

#### 2.3.3 HPLC determination

Preparation of reference solutions: appropriate amounts of PA and PB reference standards were accurately weighed and placed into 10 mL brown volumetric flasks. The standards were dissolved and diluted to volume with methanol, and then filtered through a 0.45 μm microporous membrane to obtain praeruptorin A and praeruptorin B reference solutions at concentrations of 0.406 mg·mL^−1^ and 0.402 mg·mL^−1^, respectively.

Preparation of sample solutions: Qianhu sample powder, passed through a No. 3 sieve, was accurately weighed at 0.5 g and placed into a 50 mL centrifuge tube. Methanol (25 mL) was accurately added, followed by ultrasonic extraction at 480 W and 40 kHz for 15 min. The extract was filtered through a 0.45 μm microporous membrane to obtain the sample solution.

Chromatographic conditions: the analysis was performed on an Agilent SB-C18 column (250 mm × 4.6 mm, 5 μm). The mobile phase was water–methanol (25:75, v/v). The column temperature was maintained at 35 °C, the flow rate was 1.0 mL·min^−1^, the detection wavelength was 321 nm, and the injection volume was 10 μL.

Method validation: The HPLC method was validated with respect to linearity, recovery, precision, and specificity. The calibration curves showed good linearity over the ranges of 50.00–500.00 *μ*g/mL for PA and 4.00–125.00 *μ*g/mL for PB, with correlation coefficients (*r*) of 0.9993 and 0.9997, respectively. The regression equations were *Y* = 14.17X − 6.08 for PA and *Y* = 27.96X − 17.63 for PB. The average recoveries were 99.742% (RSD = 1.165%, *n* = 6) for PA and 99.318% (RSD = 1.510%, *n* = 6) for PB. Intra-day and inter-day precision (RSD) were both less than 2.0%. Specificity was confirmed by the consistent retention times of sample peaks with those of the corresponding standards. These results indicate that the method exhibits good accuracy and reliability.

### 2.4 Data processing

#### 2.4.1 Qualitative analysis of aroma components and ROAV analysis

A mixed standard solution containing six ketones, namely 2-butanone, 2-pentanone, 2-hexanone, 2-heptanone, 2-octanone, and 2-nonanone, was analyzed to establish calibration curves between retention time and retention index. The retention index of each target compound was then calculated according to its retention time. Volatile flavor compounds in the samples were qualitatively identified by searching and matching against the GC retention index database (NIST 2020) and the IMS drift time database built into VOCal software. Fingerprint profiles were compared using the Gallery Plot plug-in to visualize differences in volatile components among Qianhu samples from different origins. Principal component analysis was performed using Origin 2022. The ROAV was used to evaluate the contribution of differential volatile compounds to the overall aroma of Qianhu from geo-authentic and other production areas. The compound contributing most to the aroma was defined as ROAV_max_ = 100, and the ROAV of other compounds was calculated as follows: ROAV_i_ = *C_i_*/*C*_max_ × *T_i_*/*T*_max_ × 100, where *C_i_* represents the relative percentage content (%) of volatile compound *i*, *T_i_* represents the corresponding odor threshold of compound *i* (mg·kg^−1^); *C*_max_ represents the relative percentage content (%) of the compound with the greatest aroma contribution, and *T*_max_ represents its corresponding odor threshold (mg·kg^−1^). A higher ROAV indicates a greater contribution to the overall aroma profile of the sample.

#### 2.4.2 High-throughput sequencing data analysis

Raw paired-end sequencing reads were quality-controlled using fastp (Chen et al., 2018) and merged using FLASH (Magoc and Salzberg, 2011). The optimized sequences obtained after quality control and merging were denoised using the DADA2 plug-in (Edgar, 2013). Data quality was evaluated by counting the number of sequences retained at each stage of data processing (Gamarra et al., 2010; Haas et al., 2011).

All data analyses were performed on the Majorbio Cloud Platform.^1^ Specifically, R software version 3.3.1 was used to generate principal coordinates analysis (PCoA) plots at the genus level. Community composition plots were generated at both the phylum and genus levels. The top 20 genera with the highest total abundance were selected to construct community heatmaps, and multiple-group comparison analysis was performed at the genus level.

#### 2.4.3 Correlation analysis

Spearman’s rank correlation analysis was performed to evaluate the relationships among key microbial taxa, volatile compounds, and major active constituents. This non-parametric method was selected because the microbial relative abundance data did not meet the assumptions of normal distribution (Shapiro–Wilk test, *p* < 0.05). To account for the large number of pairwise comparisons, the false discovery rate (FDR) was controlled using the Benjamini–Hochberg method, and only correlations with an adjusted *p* < 0.05 were considered statistically significant.

Data were statistically processed using Excel. Spearman correlation analyses were performed using SPSS 26, and figures were generated using Origin 2022.

## 3 Results

### 3.1 GC-IMS data analysis of Qianhu from different origins

#### 3.1.1 Qualitative analysis of aroma components in Qianhu

A total of 122 volatile components were detected by GC-IMS. These compounds were numbered and identified using the NIST and IMS databases, and 69 volatile components were identified (Table 2). Entries in the “Substance” column represented solely by numbers refer to compounds that produced detectable peaks in the GC-IMS spectra but could not be identified (i.e., did not match any known substances); these numbers serve as temporary identifiers. These included 15 aldehydes, 12 alkenes, 12 alcohols, 8 ketones, 7 esters, 3 thioethers, 3 hydrocarbons, 2 organic acids, 1 amide, 1 terpene, 1 nitrile, 1 epoxide, 1 furan, and 2 other compounds. Among the identified volatile components, aldehydes were the most abundant, accounting for 21.73% of all identified compounds, followed by alkenes and alcohols, each accounting for 17.39%. Together, these three classes accounted for more than 56.52% of all identified compounds.

**Table 2:** Volatile components in *Peucedanum praeruptorum*.

No.	Substance	Compound types	CAS	Chemical formula	Relative molecular mass	Retention index	Retention time/min	IMS
1	Acetic acid-M	Organic acids	C64197	C_2_H_4_O_2_	60.1	1,468.8	918.206	1.05504
2	Camphor	Ketones	C76222	C_10_H_16_O	152.2	1,569.8	1,135.782	1.46716
3	Acetic acid-D	Organic acids	C64197	C_2_H_4_O_2_	60.1	1,469.3	919.099	1.15772
4	Octanal-M	Aldehydes	C124130	C_8_H_16_O	128.2	1,302.2	648.343	1.40732
5	Octanal-D	Aldehydes	C124130	C_8_H_16_O	128.2	1,301.7	647.612	1.81647
6	2-Butanone, 3-hydroxy-M	Ketones	C513860	C_4_H_8_O_2_	88.1	1,295.6	639.507	1.06223
7	2-Butanone, 3-hydroxy-D	Ketones	C513860	C_4_H_8_O_2_	88.1	1,298.3	643.122	1.32668
8	(+)-limonene-D	Alkenes	C138863	C_10_H_16_	136.2	1,196.4	479.887	1.65739
9	1 -hexanol-M	Alcohols	C111273	C_6_H_14_O	102.2	1,366.7	741.182	1.32746
10	1 -hexanol-D	Alcohols	C111273	C_6_H_14_O	102.2	1,367	741.6	1.64033
11	(E)-2-Heptenal-M	Aldehydes	C18829555	C_7_H_12_O	112.2	1,335.2	694.227	1.25413
12	(E)-2-Heptenal-D	Aldehydes	C18829555	C_7_H_12_O	112.2	1,333.5	691.749	1.66614
13	7	*	*	*	*	1,329.5	686.114	1.35243
14	alpha-terpinolene-M	Alkenes	C586629	C_10_H_16_	136.2	1,289.6	629.334	1.30196
15	alpha-terpinolene-D	Alkenes	C586629	C_10_H_16_	136.2	1,285.3	621.592	1.74539
16	18	*	*	*	*	1,288.7	627.758	1.18893
17	4	*	*	*	*	1,126	380.35	1.63162
18	13	*	*	*	*	989.6	252.345	1.44973
19	14	*	*	*	*	930.2	214.504	1.40897
20	15	*	*	*	*	931.5	215.278	1.3384
21	16	*	*	*	*	835.7	165.73	1.1454
22	11	*	*	*	*	1,096.2	343.616	1.72202
23	35	*	*	*	*	1,337.6	697.737	1.83996
24	1	*	*	*	*	1,337.6	697.737	1.89862
25	Linalool oxide	Terpene alcohols	C1365191	C_10_H_18_O_2_	170.3	1,472.3	925.059	1.25886
26	benzaldehyde	Aldehydes	C100527	C_7_H_6_O	106.1	1,503.8	988.463	1.15323
27	Acetic acid ethyl ester	Esters	C141786	C_4_H_8_O_2_	88.1	900.1	197.56	1.33476
28	2-Butanone	Ketones	C78933	C_4_H_8_O	72.1	927.8	213.062	1.24325
29	28	*	*	*	*	987.2	250.577	1.35821
30	Heptaldehyde-D	Aldehydes	C111717	C_7_H_14_O	114.2	1,186.7	466.624	1.68608
31	Heptaldehyde-M	Aldehydes	C111717	C_7_H_14_O	114.2	1,185.9	465.497	1.33536
32	9	*	*	*	*	1,089.6	335.987	1.559
33	1-Pentanol-M	Alcohols	C71410	C_5_H_12_O	88.1	1,261.1	579.264	1.25088
34	1-Pentanol-D	Alcohols	C71410	C_5_H_12_O	88.1	1,261.8	580.566	1.50914
35	23	*	*	*	*	916.7	206.75	1.1207
36	6-methyl-5-hepten-2-one	Ketones	C110930	C_8_H_14_O	126.2	1,349	714.388	1.17569
37	Dipropyl disulfide	Thioethers	C629196	C_6_H_14_S_2_	150.3	1,367.4	742.253	1.25208
38	(E)-2-Octenal	Aldehydes	C2548870	C_8_H_14_O	126.2	1,396.5	788.472	1.33732
39	(E)-beta-caryophyllene-M	Alkenes	C87445	C_15_H_24_	204.4	1,552.6	1,095.545	1.42834
40	45	*	*	*	*	1,338	698.226	1.31238
41	3-nonanone	Ketones	C925780	C_9_H_18_O	142.2	1,344.2	707.26	1.37273
42	46	*	*	*	*	1,299.3	644.393	1.51828
43	47	*	*	*	*	1,299.6	644.803	1.58938
44	3-Methyl-3-buten-1-ol	Enols	C763326	C_5_H_10_O	86.1	1,249.6	560.252	1.16165
45	32	*	*	*	*	1,241.7	547.518	1.28384
46	8	*	*	*	*	1,090.5	336.967	1.27474
47	3-Methyl-2-butenal	Aldehydes	C107868	C_5_H_8_O	84.1	1,203.8	490.422	1.09096
48	2	*	*	*	*	1,204.3	491.172	1.21509
49	(E)-2-hexenal-D	Aldehydes	C6728263	C_6_H_10_O	98.1	1,223.4	519.157	1.50707
50	(E)-2-hexenal-M	Aldehydes	C6728263	C_6_H_10_O	98.1	1,223.9	519.907	1.18187
51	1-Butanol, 3-methyl-M	Alcohols	C123513	C_5_H_12_O	88.1	1,211.1	500.95	1.24661
52	1-Butanol, 3-methyl-D	Alcohols	C123513	C_5_H_12_O	88.1	1,211.3	501.167	1.49133
53	36	*	*	*	*	1,205	492.172	2.18368
54	48	*	*	*	*	1,206.4	494.171	1.6347
55	49	*	*	*	*	1,206.3	493.921	1.72736
56	19	*	*	*	*	1,185.5	464.936	1.38643
57	alpha-Terpinene-D	Alkenes	C99865	C_10_H_16_	136.2	1,176.4	451.693	1.71512
58	alpha-Terpinene-M	Alkenes	C99865	C_10_H_16_	136.2	1,176.6	451.943	1.21335
59	40	*	*	*	*	1,134.5	391.585	1.07308
60	Allyl sulfide-M	Thioethers	C592881	C_6_H_10_S	114.2	1,131.4	387.388	1.11721
61	Allyl sulfide-D	Thioethers	C592881	C_6_H_10_S	114.2	1,131.4	387.388	1.32429
62	Butanol-M	Alcohols	C71363	C_4_H_10_O	74.1	1,144.9	405.752	1.18171
63	Butanol-D	Alcohols	C71363	C_4_H_10_O	74.1	1,143.8	404.178	1.3803
64	3	*	*	*	*	1,127.8	382.666	1.21735
65	30	*	*	*	*	1,133	389.627	1.24988
66	29	*	*	*	*	1,130.7	386.559	1.29716
67	33	*	*	*	*	1,131.7	387.822	1.41874
68	34	*	*	*	*	1,132	388.183	1.5589
69	50	*	*	*	*	1,121.1	374.137	2.18092
70	43	*	*	*	*	1,095.8	343.136	1.04215
71	20	*	*	*	*	1,096.1	343.545	1.18141
72	37	*	*	*	*	1,090	336.434	1.31772
73	38	*	*	*	*	1,088.8	335.052	1.3677
74	10	*	*	*	*	1,095.6	342.953	1.41314
75	5	*	*	*	*	1,075.3	322.213	1.21321
76	2,3-pentadione	Ketones	C600146	C_5_H_8_O_2_	100.1	1,063.5	311.547	1.21473
77	21	*	*	*	*	1,094.6	341.767	1.48735
78	27	*	*	*	*	1,056.1	305.028	1.04661
79	6	*	*	*	*	1,051.5	301.078	1.20412
80	31	*	*	*	*	1,043.2	293.967	1.14809
81	alpha-Pinene	Alkenes	C80568	C_10_H_16_	136.2	1,027.8	281.326	1.67516
82	39	*	*	*	*	1,105.4	354.606	1.58277
83	22	*	*	*	*	1,014.1	270.565	1.10989
84	4-Methyl-2-pentanone-M	Ketones	C108101	C_6_H_12_O	100.2	1,013.5	270.076	1.18012
85	4-Methyl-2-pentanone-D	Ketones	C108101	C_6_H_12_O	100.2	1,015.7	271.835	1.48852
86	acrylonitrile	Nitriles	C107131	C_3_H_3_N	53.1	1,003.8	262.745	1.08161
87	2-Methylbutanoic acid, methyl ester	Esters	C868575	C_6_H_12_O_2_	116.2	1,001.1	260.727	1.19459
88	12	*	*	*	*	989.8	252.454	1.17682
89	51	*	*	*	*	982.8	247.611	1.0778
90	44	*	*	*	*	962.5	234.293	1.07907
91	Acetic acid propyl ester	Esters	C109604	C_5_H_10_O_2_	102.1	978	244.382	1.16031
92	52	*	*	*	*	967.3	237.32	1.18062
93	24	*	*	*	*	892.6	193.576	1.13626
94	42	*	*	*	*	875.9	184.916	1.04621
95	17	*	*	*	*	893.2	193.893	1.28099
96	25	*	*	*	*	861.1	177.625	1.15211
97	methyl acetate	Esters	C79209	C_3_H_6_O_2_	74.1	863.4	178.764	1.19466
98	Butanal-D	Aldehydes	C123728	C_4_H_8_O	72.1	843	169.037	1.27975
99	Butanal-M	Aldehydes	C123728	C_4_H_8_O	72.1	853.8	174.107	1.11619
100	26	*	*	*	*	817.7	157.757	1.09066
101	41	*	*	*	*	773.7	139.933	1.22
102	beta-cyclocitral	Aldehydes	C432257	C_10_H_16_O	152.2	1,600.1	1,210.671	1.31233
103	beta-ocimene	Alkenes	C13877913	C_10_H_16_	136.2	1,242.2	548.346	1.69816
104	Citronellyl acetate	Esters	C150845	C_12_H_22_O_2_	198.3	1,656.3	1,362.85	1.46821
105	(R/S)-linalool	Terpene alcohols	C78706	C_10_H_18_O	154.3	1,557	1,105.53	1.21722
106	Ethanol	Alcohols	C64175	C_2_H_6_O	46.1	933.8	216.589	1.12984
107	beta-myrcene-D	Alkenes	C123353	C_10_H_16_	136.2	1,145.8	406.924	1.71745
108	beta-Myrcene-M	Alkenes	C123353	C_10_H_16_	136.2	1,146.9	408.468	1.29114
109	Ethyl formate-M	Esters	C109944	C_3_H_6_O_2_	74.1	838.1	166.811	1.07374
110	Ethyl formate-D	Esters	C109944	C_3_H_6_O_2_	74.1	843.1	169.094	1.21171
111	Methylcyclohexane	Hydrocarbons	C108872	C_7_H_14_	98.2	788.4	145.67	1.13111
112	ethylcyclopentane	Hydrocarbons	C1640897	C_7_H_14_	98.2	773.4	139.807	1.113
113	Acetaldehyde	Aldehydes	C75070	C_2_H_4_O	44.1	738	126.937	0.95507
114	2-pentyl furan	Furans	C3777693	C_9_H_14_O	138.2	1,240.1	544.942	1.24549
115	53	*	*	*	*	1,108.9	358.865	2.10292
116	gamma-Terpinene	Alkenes	C99854	C_10_H_16_	136.2	1,250.5	561.737	1.70107
117	Propanol-M	Alcohols	C71238	C_3_H_8_O	60.1	1,046.3	296.603	1.11604
118	Propanol-D	Alcohols	C71238	C_3_H_8_O	60.1	1,044.7	295.246	1.25612
119	delta-3-Carene-T	Alkenes	C13466789	C_10_H_16_	136.2	1,163.1	431.626	2.16696
120	delta-3-Carene-M	Alkenes	C13466789	C_10_H_16_	136.2	1,164.2	433.361	1.68683
121	(E)-beta-Farnesene	Alkenes	C18794848	C_15_H_24_	204.4	1,689.4	1,461.27	1.43182
122	(+)-limonene-M	Alkenes	C138863	C_10_H_16_	136.2	1,195.6	478.799	1.30233

#### 3.1.2 Gallery plot fingerprint analysis of aroma components in Qianhu

To further investigate differences and variation patterns in aroma components among Qianhu samples from different origins, three parallel samples from each origin were selected to construct Gallery Plot fingerprints of representative aroma components (Figure 1). In the figure, sample names are shown on the far right, while the identified aroma components are listed at the bottom. Numerical codes indicate some unidentified compounds. The aroma components and their relative distributions varied among Qianhu samples from different origins, indicating that aroma component profiles showed origin-specific characteristics and may serve as potential markers for origin traceability.

**Figure 1:**
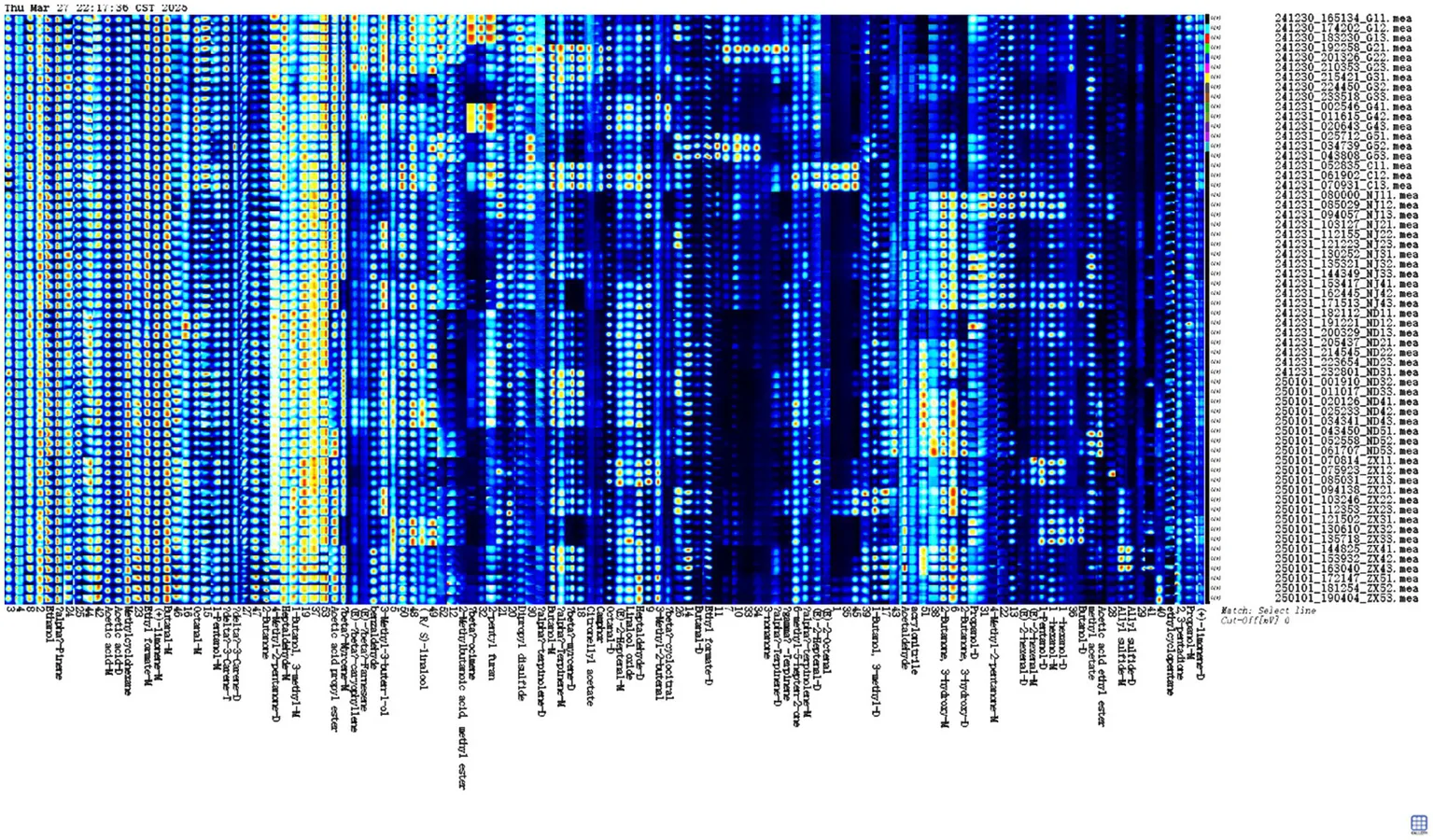
Volatile component fingerprints of Peucedani Radix from different producing regions.

As shown in Figure 1, the content of (E)-2-hexenal-D was higher in NJ1 than in samples from other origins; propanol-M was higher in ND1; allyl sulfide-M and allyl sulfide-D were higher in ZX4; 3-nonanone, alpha-terpinene-D, and gamma-terpinene were higher in G2; beta-cyclocitral was higher in G4; butanal-D and ethyl formate-D were higher in G5; and (E)-2-octenal was higher in C1.

Compared with samples from other origins, Anhui samples showed higher responses of acrylonitrile, 2-butanone, 3-hydroxy-M, 2-butanone, 3-hydroxy-D, and propanol-D. In particular, samples from Nanji Village, Ningguo City, Anhui Province showed a higher response of 4-methyl-2-pentanone-M.

#### 3.1.3 Principal component analysis

PCA was performed on the volatile components of Qianhu samples from different origins, yielding a three-dimensional PCA score plot (Figure 2) that allowed visual differentiation among samples from different production areas.

**Figure 2:**
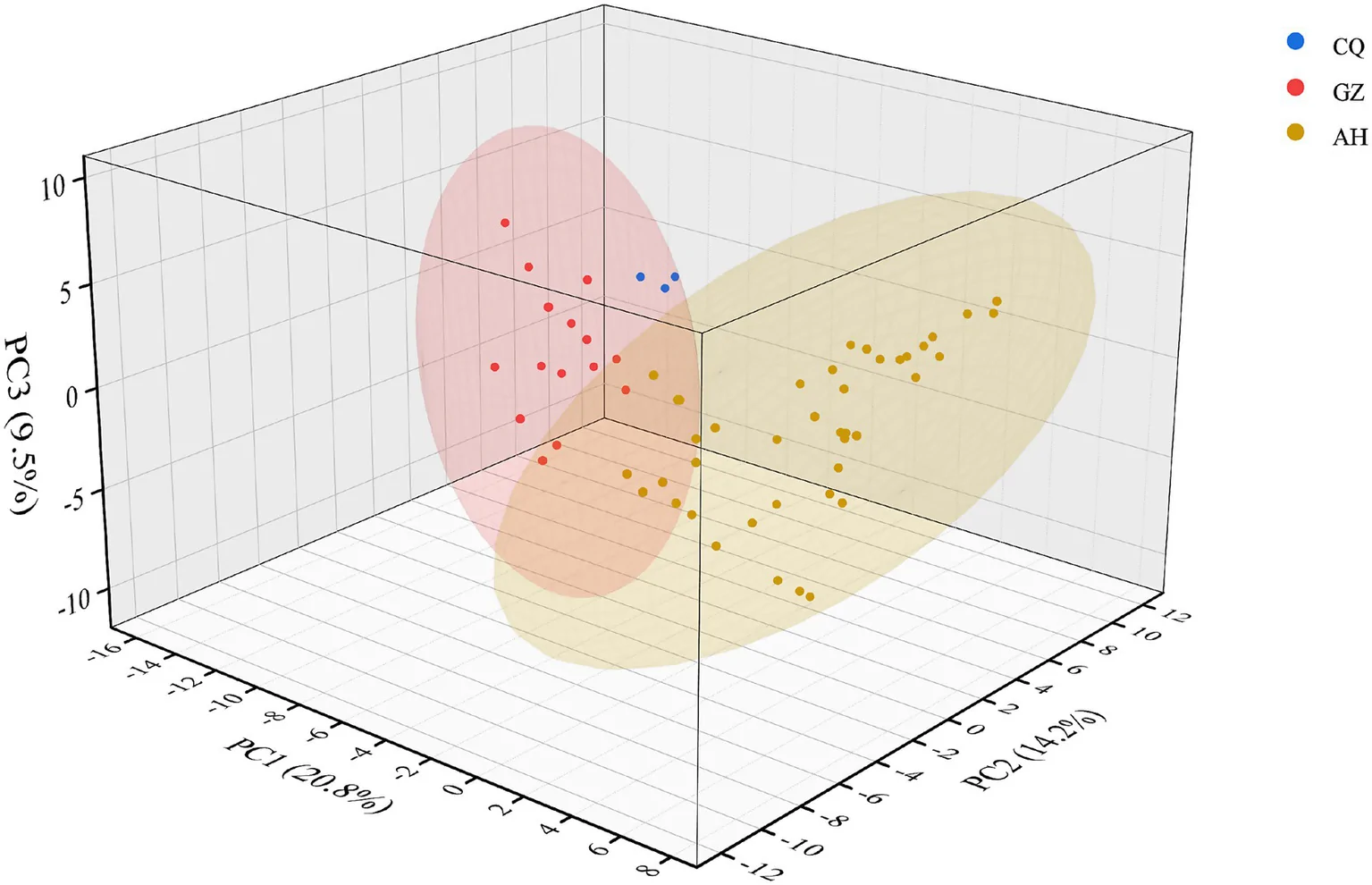
3D l diagram for principal component analysis of Peucedani Radix from different producing regions.

As shown in Figure 2, the contribution rates of PC1, PC2, and PC3 were 20.8, 14.2, and 9.5%, respectively, with a cumulative contribution rate of 44.5%. Samples from the geo-authentic production area, Ningguo, showed a certain separation trend from samples from other regions, including Guizhou and Chongqing, in the principal component space. This result indicates that geographical origin has a certain influence on the volatile components of Qianhu, which may be related to factors such as latitude, soil conditions, and cultivation practices.

#### 3.1.4 ROAV analysis

The contribution of volatile components to sample flavor is jointly determined by their contents and odor thresholds. A higher ROAV indicates a greater contribution to the overall flavor of the sample (Feng et al., 2024). To further clarify the differential volatile components distinguishing Qianhu from geo-authentic and other production areas, ROAV was used to evaluate the contribution of each differential volatile component to the overall aroma of each sample. Because 2-methylbutanoic acid, methyl ester had a relatively low odor threshold and high peak intensity in the 20 samples, it contributed the most to the overall aroma. Therefore, the ROAVmax of 2-methylbutanoic acid, methyl ester, was defined as 100, and the ROAV values of the remaining components were normalized accordingly, as shown in Table 3.

**Table 3:** Volatile components in *Peucedanum praeruptorum* from different producing regions.

No.	Substance	Odor threshold (mg/kg)	ROAV (mg/kg)				
			GZ	CQ	NJ	ND	ZX
1	2-Methylbutanoic acid, methyl ester	0.00025	100.00	100.00	100.00	100.00	100.00
2	beta-cyclocitral	0.003	3.19	0.58	1.60	8.08	2.67
3	Camphor	4.6	<0.01	<0.01	<0.01	<0.01	<0.01
4	(E)-beta-caryophyllene	0.064	0.12	0.06	0.19	0.39	0.10
5	benzaldehyde	0.35	0.02	0.02	0.05	0.07	0.05
6	Acetic acid-M	0.013	3.08	2.24	8.76	14.34	6.98
7	Acetic acid-D	0.013	0.71	0.56	2.42	3.97	1.94
8	Linalool oxide	ND	/	/	/	/	/
9	Dipropyl disulfide	0.13	0.04	0.02	0.09	0.16	0.06
10	1 -hexanol-M	0.5	0.01	0.01	0.07	0.10	0.06
11	Octanal-M	0.021	1.47	0.93	3.58	6.32	2.93
12	alpha-Terpinene-D	0.08	0.51	0.24	1.27	2.14	1.00
13	delta-3-Carene-T	0.77	0.03	0.03	0.08	0.13	0.07
14	2,3-pentadione	0.01	3.94	2.33	13.18	21.17	12.22
15	alpha-Pinene	0.014	3.32	2.41	9.60	15.65	7.70
16	(+)-limonene-D	0.2	0.07	0.05	0.21	0.36	0.16
17	beta-myrcene-D	0.015	0.85	0.14	1.95	3.38	1.62
18	gamma-Terpinene	1	0.01	0.01	0.02	0.03	0.02
19	Heptaldehyde-D	0.0028	6.56	4.34	16.98	28.71	13.69
20	Heptaldehyde-M	0.0028	7.23	5.33	26.62	44.19	23.68
21	Allyl sulfide-M	0.001	5.61	3.65	27.92	42.30	31.36
22	(E)-2-hexenal-D	0.0887	0.13	0.05	0.30	0.42	0.29
23	Acetic acid propyl ester	1	0.05	0.04	0.12	0.17	0.11
24	Ethanol	950	<0.01	<0.01	<0.01	<0.01	<0.01
25	Butanal-D	0.1	1.24	0.88	3.58	5.82	2.89
26	2-Butanone, 3-hydroxy-M	8	<0.01	<0.01	0.01	0.02	0.01
27	1-Pentanol-M	0.153	0.14	0.07	0.37	0.57	0.34
28	(E)-2-Heptenal-M	0.25	0.03	0.02	0.10	0.16	0.09
29	alpha-terpinolene-M	0.2	0.07	0.03	0.28	0.48	0.19
30	2-Butanone	1.3	0.03	0.03	0.11	0.18	0.09
31	methyl acetate	1.5	0.03	0.02	0.08	0.13	0.05
32	Ethyl formate-M	90.9	<0.01	<0.01	<0.01	<0.01	<0.01
33	Methylcyclohexane	0.6	0.05	0.05	0.19	0.30	0.15
34	4-Methyl-2-pentanone-M	0.7	0.02	0.01	0.06	0.09	0.04
35	acrylonitrile	18.6	<0.01	<0.01	<0.01	0.01	<0.01
36	1-Butanol, 3-methyl-M	0.0061	2.69	2.25	10.39	17.25	8.42
37	alpha-Terpinene-M	0.08	0.08	0.02	0.14	0.24	0.11
38	Octanal-D	0.021	1.26	0.88	3.11	5.26	2.49
39	2-Butanone, 3-hydroxy-D	8	<0.01	<0.01	0.01	0.01	0.01
40	1 -hexanol-D	0.5	<0.01	<0.01	0.01	0.01	0.01
41	(E)-2-Heptenal-D	0.25	<0.01	<0.01	0.01	0.02	0.01
42	alpha-terpinolene-D	0.2	<0.01	<0.01	0.01	0.02	0.01
43	Citronellyl acetate	1	<0.01	<0.01	<0.01	<0.01	<0.01
44	(R/S)-linalool	0.0024	0.74	0.51	2.22	3.91	1.71
45	Acetic acid ethyl ester	5.36	<0.01	<0.01	0.01	0.01	0.01
46	1-Pentanol-D	0.153	0.01	0.01	0.05	0.06	0.06
47	(E)-beta-Farnesene	ND	/	/	/	/	/
48	3-nonanone	ND	/	/	/	/	/
49	3-Methyl-3-buten-1-ol	ND	/	/	/	/	/
50	6-methyl-5-hepten-2-one	0.3	0.01	<0.01	0.02	0.04	0.02
51	1-Butanol, 3-methyl-D	0.0061	0.88	2.02	8.83	14.76	7.48
52	3-Methyl-2-butenal	ND	/	/	/	/	/
53	Butanol-M	0.48	0.10	0.04	0.25	0.42	0.20
54	Butanol-D	0.48	<0.01	<0.01	0.01	0.02	0.01
55	Allyl sulfide-D	0.001	5.10	2.78	33.49	35.80	51.42
56	Ethyl formate-D	90.9	90.9	<0.01	<0.01	<0.01	<0.01
57	Butanal-M	0.03	0.09	0.11	0.19	0.09	0.03
58	ethylcyclopentane	ND	/	/	/	/	/
59	(E)-2-Octenal	0.012	0.06	0.03	0.21	0.36	0.16
60	beta-ocimene	ND	/	/	/	/	/
61	(E)-2-hexenal-M	0.0887	0.02	0.01	0.05	0.06	0.05
62	delta-3-Carene-M	0.77	0.02	0.01	0.05	0.08	0.04
63	beta-Myrcene-M	0.015	2.05	0.88	4.96	8.52	3.88
64	4-Methyl-2-pentanone-D	0.7	0.01	<0.01	0.01	0.01	0.01
65	Acetaldehyde	0.0251	0.58	0.50	2.65	4.16	2.35
66	2-pentyl furan	0.019	0.44	0.30	1.09	1.77	0.84
67	Propanol-M	0.24	0.03	0.03	0.14	0.21	0.10
68	Propanol-D	0.24	0.06	0.06	0.17	0.27	0.13
69	(+)-limonene-M	0.2	0.28	0.20	0.78	1.29	0.62

As shown in Table 3, the ROAV values of 2-methylbutanoic acid, methyl ester; allyl sulfide-D; allyl sulfide-M; heptaldehyde-M; heptaldehyde-D; 2,3-pentadione; 1-butanol, 3-methyl-M; alpha-pinene; 1-butanol, 3-methyl-D; and acetic acid-M were greater than 1 in Qianhu from all origins. These compounds can be considered key volatile flavor compounds in Qianhu. Moreover, the ROAV values of these ten aroma components in Ning Qianhu were higher than those in samples from Chongqing and Guizhou, indicating that Ning Qianhu had a stronger aroma. Among these compounds, aldehydes and thioethers showed relatively high ROAV values in Ning Qianhu, suggesting that they may be important contributors to its characteristic aroma. Heptaldehyde-M and heptaldehyde-D, as representative aldehydes, generally exhibited high ROAV values and may be associated with lipid oxidation or amino acid metabolism pathways in Qianhu. Diallyl sulfide compounds are important sulfur-containing flavor substances, usually associated with pungent, onion-like, and garlic-like odor characteristics, and may influence the aroma style of Qianhu to some extent. Alpha-pinene, a representative terpene compound widely present in Apiaceae plants, has a pine-like fresh aroma and reflects the contribution of terpenes to the aroma profile of Ning Qianhu.

In summary, these 10 volatile components with ROAV values greater than 1 contributed significantly to aroma across the five production areas and constituted the core flavor components of Qianhu.

### 3.2 Diversity analysis of rhizosphere microorganisms from different origins

#### 3.2.1 Rarefaction curve analysis

Using QIIME software, the sequence numbers of each sample in the amplicon sequence variants (ASV) abundance matrix were randomly subsampled at different sequencing depths to generate rarefaction curves. The Shannon index rarefaction curves of rhizosphere bacteria and fungi from different production regions of Qianhu are shown in Figures 3A,B, respectively. As illustrated in these figures, with the increase of sampling reads (*x*-axis), most curves rose rapidly at the initial stage, indicating that the detected species diversity increased quickly with the increase of sampled sequences at the early stage of sampling. As sequencing depth increased, the slopes of the curves gradually decreased, and most curves gradually leveled off, suggesting that when sampling reached a certain depth, even further increases in the number of sampled reads did not significantly increase the species diversity index (Shannon index). This indicates that the majority of species in the samples had been covered, and that the sequencing depth was sufficient to reflect the true species diversity. These results demonstrated that the sequencing data obtained from different samples could comprehensively reflect the overall species information, and that the obtained sequencing results were suitable for the diversity analysis of rhizosphere microbial communities in Qianhu.

**Figure 3:**
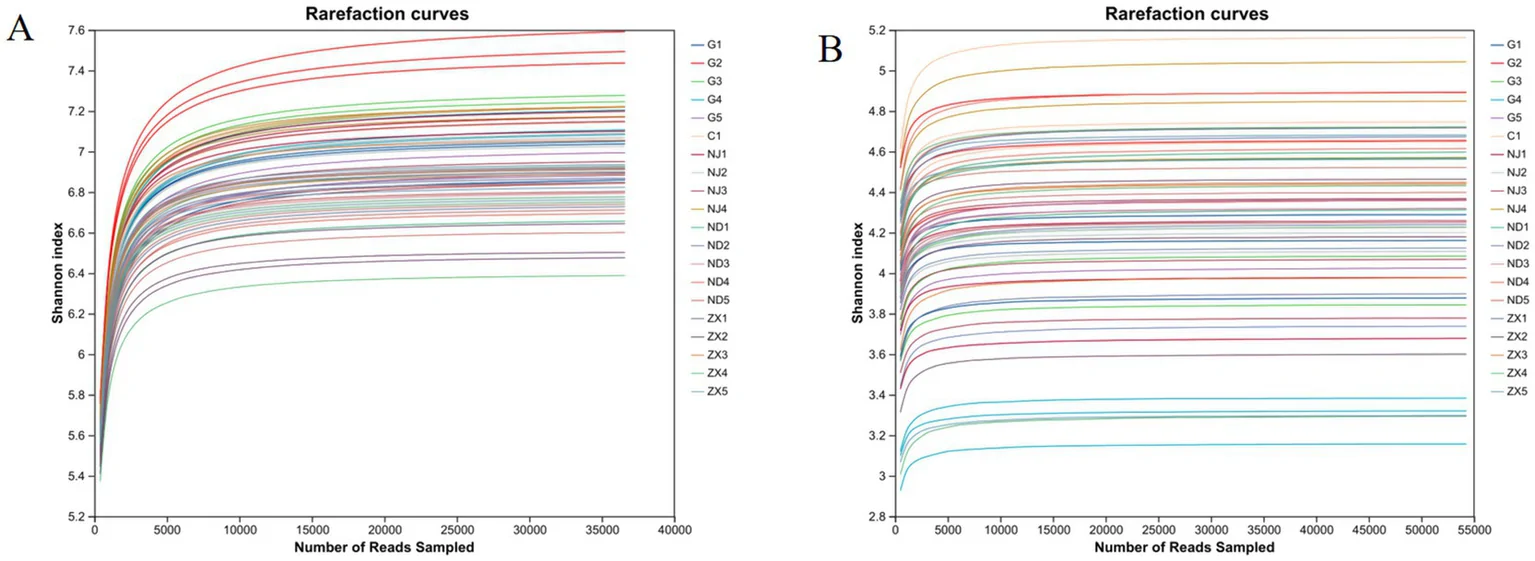
Rarefaction curves of rhizosphere soil microbial communities associated with *Peucedanum praeruptorum*. **(A)** Bacteria; **(B)** Fungi.

#### 3.2.2 Alpha diversity analysis

Alpha diversity analysis primarily evaluates the richness and diversity of microbial communities in environmental samples through multiple diversity indices. The ACE index is a metric for estimating species richness that accounts for both abundant and rare species in a sample; a higher value indicates potentially greater species richness. The Chao index is also used to estimate species richness, primarily based on low-abundance species in the sample; it is widely applied in assessing community species richness, with higher values indicating greater species richness. The Shannon index is a diversity index that comprehensively considers both species richness and evenness; a higher value indicates greater species diversity and more even species distribution within the community. The Simpson index measures diversity, with lower values indicating higher community diversity, and it more strongly reflects the influence of dominant species on the community. The Pielou_e index, or evenness index, reflects the uniformity of species individual distribution within a community; values closer to 1 indicate more even species distribution.

The Alpha diversity indices of rhizosphere bacteria from different Qianhu samples are shown in Table 4. The ACE and Chao indices of G2 were higher than those of other groups, followed by G3, G4, C1, NJ1, G5, NJ4, NJ3, and NJ2, suggesting that the rhizosphere bacterial species richness in these regions was relatively high. The Shannon indices of G2, G3, NJ4, C1, and NJ1 were higher, indicating relatively abundant and evenly distributed rhizosphere bacterial species diversity. The Simpson indices of C2 and NJ4 were lower, suggesting higher community diversity, meaning that dominant species had a relatively smaller impact on community structure in these samples. In contrast, samples such as ZX4, ND1, and G5 showed higher Simpson indices, indicating lower community diversity, meaning that dominant species had a relatively greater impact on community structure. The Pielou_e indices of most samples ranged from 0.8 to 0.9, indicating that the rhizosphere bacterial species distribution was relatively even across most samples.

**Table 4:** Table of alpha diversity indices of rhizosphere soil bacterial in different samples.

Group	ACE index	Chao index	Shannon index	Simpson index	Pielou_e index
C1	2,892.48 ± 292.91	2,867.72 ± 294.26	7.17 ± 0.04	0.002 ± 0.00005	0.907 ± 0.00
G1	2,481.90 ± 129.61	2,470.33 ± 129.52	6.98 ± 0.11	0.002 ± 0.00051	0.898 ± 0.02
G2	4,215.67 ± 531.63	4,075.15 ± 512.84	7.51 ± 0.08	0.001 ± 0.00006	0.913 ± 0.00
G3	3,106.92 ± 257.06	3,067.82 ± 235.37	7.25 ± 0.03	0.002 ± 0.00023	0.907 ± 0.01
G4	3,102.42 ± 205.21	3,055.55 ± 194.57	7.13 ± 0.06	0.002 ± 0.00020	0.896 ± 0.00
G5	2,653.88 ± 294.27	2,610.48 ± 264.78	6.92 ± 0.06	0.003 ± 0.00057	0.886 ± 0.01
NJ1	2,719.66 ± 346.84	2,685.40 ± 328.17	7.16 ± 0.05	0.002 ± 0.00015	0.911 ± 0.01
NJ2	2,431.83 ± 188.92	2,415.32 ± 181.10	7.06 ± 0.03	0.002 ± 0.00009	0.909 ± 0.01
NJ3	2,461.32 ± 210.10	2,432.10 ± 200.52	6.98 ± 0.16	0.002 ± 0.00128	0.898 ± 0.02
NJ4	2,639.55 ± 229.86	2,627.85 ± 235.50	7.20 ± 0.03	0.001 ± 0.00011	0.918 ± 0.01
ND1	1,914.70 ± 180.16	1,906.28 ± 178.03	6.83 ± 0.15	0.003 ± 0.00071	0.905 ± 0.01
ND2	1,725.40 ± 29.66	1,719.45 ± 29.80	6.85 ± 0.05	0.002 ± 0.00020	0.920 ± 0.00
ND3	1,717.53 ± 61.22	1,715.40 ± 62.13	6.88 ± 0.07	0.002 ± 0.00030	0.923 ± 0.01
ND4	1,731.88 ± 71.07	1,724.70 ± 70.43	6.78 ± 0.08	0.002 ± 0.00051	0.910 ± 0.01
ND5	1,661.44 ± 62.85	1,656.33 ± 65.01	6.68 ± 0.07	0.003 ± 0.00053	0.902 ± 0.01
ZX1	1,760.23 ± 63.62	1,755.47 ± 62.99	6.84 ± 0.10	0.002 ± 0.00048	0.916 ± 0.01
ZX2	1,468.05 ± 127.96	1,465.38 ± 126.30	6.54 ± 0.09	0.003 ± 0.00042	0.898 ± 0.01
ZX3	1,858.71 ± 122.82	1,854.18 ± 120.61	6.95 ± 0.09	0.002 ± 0.00024	0.924 ± 0.00
ZX4	1,559.19 ± 126.58	1,553.51 ± 124.77	6.64 ± 0.22	0.003 ± 0.00092	0.904 ± 0.02
ZX5	1,690.57 ± 83.50	1,686.55 ± 85.03	6.84 ± 0.09	0.002 ± 0.00018	0.921 ± 0.01

The Alpha diversity indices of rhizosphere fungi from different Qianhu samples are shown in Table 5. The ACE and Chao indices of ND4, NJ4, and ND1 were higher than those of other groups, followed by ZX1 and C1, suggesting that the rhizosphere fungal species richness in these regions was relatively high. The Shannon indices of C1, NJ4, ND4, G2, and ND1 were higher, indicating relatively abundant and evenly distributed rhizosphere fungal species diversity. The Simpson indices of NJ4, G2, and ND4 were lower, suggesting higher community diversity, meaning that dominant species had a relatively smaller impact on community structure in these samples. In contrast, samples such as G4, ZX5, and ZX4 showed higher Simpson indices, indicating lower community diversity, meaning that dominant species had a relatively greater impact on community structure. The Pielou_e indices of most samples ranged from 0.6 to 0.7, indicating that the rhizosphere fungal species distribution was relatively even across most samples.

**Table 5:** Table of alpha diversity indices of rhizosphere soil fungi in different samples.

Group	ACE index	Chao index	Shannon index	Simpson index	Pielou_e index
C1	803.43 ± 90.52	802.52 ± 89.71	4.85 ± 0.27	0.028 ± 0.01	0.73 ± 0.04
G1	500.45 ± 45.18	497.76 ± 43.70	4.11 ± 0.21	0.049 ± 0.02	0.66 ± 0.04
G2	712.40 ± 25.27	704.65 ± 24.12	4.63 ± 0.27	0.027 ± 0.01	0.71 ± 0.04
G3	674.64 ± 47.87	670.52 ± 49.52	4.05 ± 0.19	0.062 ± 0.01	0.62 ± 0.02
G4	433.69 ± 8.83	432.11 ± 6.77	3.29 ± 0.12	0.155 ± 0.04	0.54 ± 0.02
G5	692.43 ± 57.86	689.46 ± 59.55	4.24 ± 0.18	0.043 ± 0.01	0.65 ± 0.02
NJ1	551.52 ± 58.39	547.22 ± 59.70	3.97 ± 0.29	0.053 ± 0.02	0.63 ± 0.04
NJ2	580.63 ± 14.85	577.06 ± 16.77	4.18 ± 0.06	0.041 ± 0.00	0.66 ± 0.01
NJ3	593.48 ± 24.91	589.59 ± 24.74	4.07 ± 0.29	0.056 ± 0.03	0.64 ± 0.04
NJ4	875.08 ± 81.16	872.01 ± 80.17	4.82 ± 0.24	0.025 ± 0.01	0.71 ± 0.03
ND1	872.97 ± 51.74	871.33 ± 51.68	4.49 ± 0.16	0.060 ± 0.03	0.66 ± 0.02
ND2	657.20 ± 54.50	652.49 ± 54.78	4.03 ± 0.26	0.055 ± 0.02	0.62 ± 0.03
ND3	756.38 ± 18.85	753.85 ± 17.91	4.31 ± 0.05	0.051 ± 0.02	0.65 ± 0.01
ND4	878.93 ± 42.46	876.02 ± 42.53	4.74 ± 0.14	0.028 ± 0.01	0.70 ± 0.02
ND5	561.23 ± 47.47	560.21 ± 46.54	4.39 ± 0.14	0.035 ± 0.01	0.69 ± 0.03
ZX1	803.73 ± 77.02	800.62 ± 77.05	4.43 ± 0.46	0.046 ± 0.03	0.66 ± 0.06
ZX2	578.92 ± 78.68	577.57 ± 78.77	4.08 ± 0.44	0.056 ± 0.03	0.64 ± 0.06
ZX3	670.71 ± 56.72	669.20 ± 56.69	4.29 ± 0.27	0.045 ± 0.022	0.66 ± 0.04
ZX4	725.43 ± 93.07	721.23 ± 94.37	4.15 ± 0.75	0.070 ± 0.070	0.63 ± 0.10
ZX5	622.37 ± 107.37	618.66 ± 107.03	4.18 ± 0.77	0.074 ± 0.091	0.65 ± 0.10

#### 3.2.3 PCoA analysis of rhizosphere microbial communities

PCoA is an unconstrained dimensionality-reduction method used to assess similarities and differences in community composition across samples.

Based on the Hellinger distance matrix, PCoA analysis of bacterial community structure at the ASV level was performed in R, and intergroup differences were tested using the Adonis method (Figure 4A). The contribution rates of PC1 and PC2 were 14.63 and 7.88%, respectively, with an *R*^2^ value of 0.2651 (*p* = 0.001). Samples from different regions showed a certain degree of separation, with the CQ and GZ groups partially overlapping with the Anhui groups. The *R*^2^ value of 0.2651 indicates that approximately 26.5% of the total variance is explained by the grouping, suggesting a weak but statistically significant differentiation among regions (*p* = 0.001).

**Figure 4:**
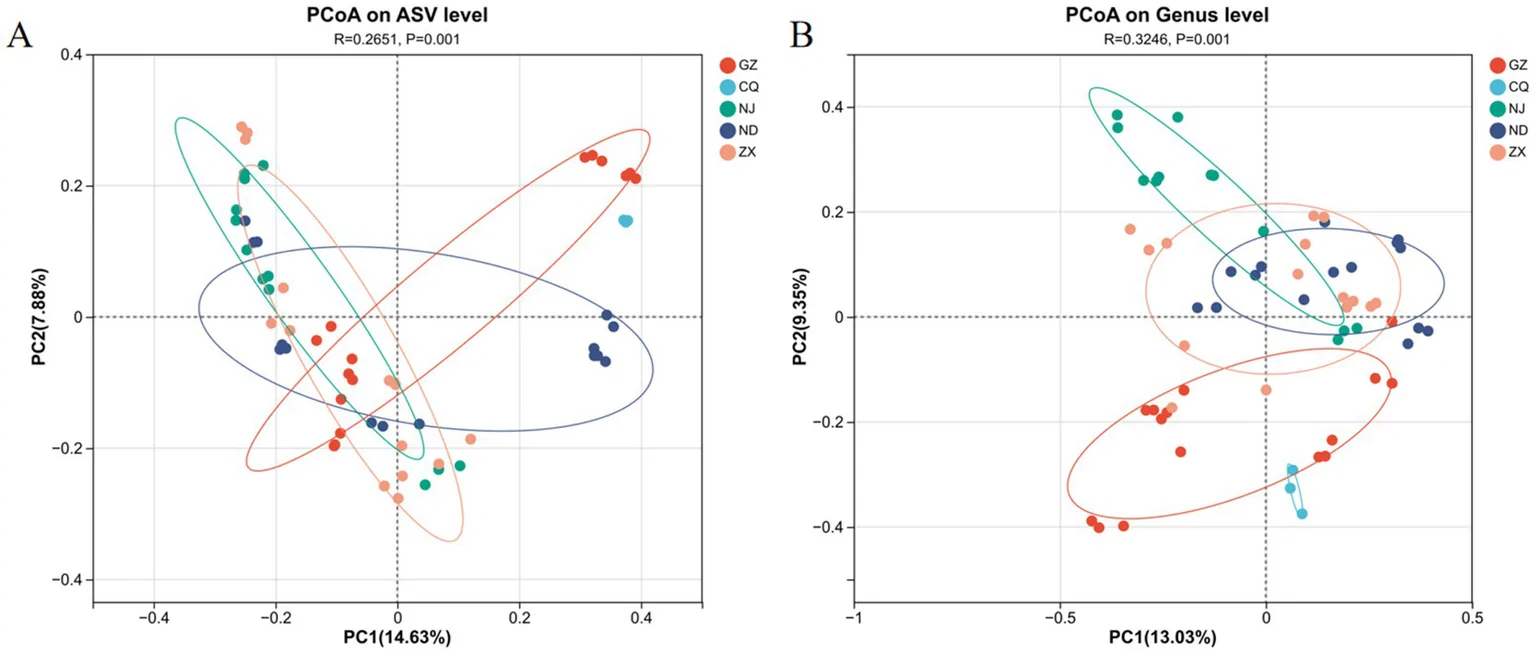
Principal coordinate analysis (PCoA) of communities in rhizosphere soil of Peucedani Radix from different producing regions. **(A)** Bacteria; **(B)** Fungi.

For fungal communities, PCoA based on the Hellinger distance matrix at the genus level was conducted in R, with intergroup differences tested using the Adonis method (Figure 4B). The contribution rates of PC1 and PC2 were 13.03 and 9.35%, respectively, with an *R*^2^ value of 0.3246 (*p* = 0.001). Samples from GZ, CQ, and Anhui showed clearer separation compared to bacterial communities, indicating more pronounced differences among the three regions. However, samples from the three Anhui locations (NJ, ND, ZX) largely overlapped, suggesting a certain degree of similarity within the geo-authentic region. The *R*^2^ value of 0.3246 indicates that approximately 32.5% of the total variance is explained by the grouping, reflecting a moderate but statistically significant differentiation (*p* = 0.001).

#### 3.2.4 Composition analysis of rhizosphere microbial communities

The composition of rhizosphere bacterial communities at the phylum and genus levels is shown in Figures 5A,B.

**Figure 5:**
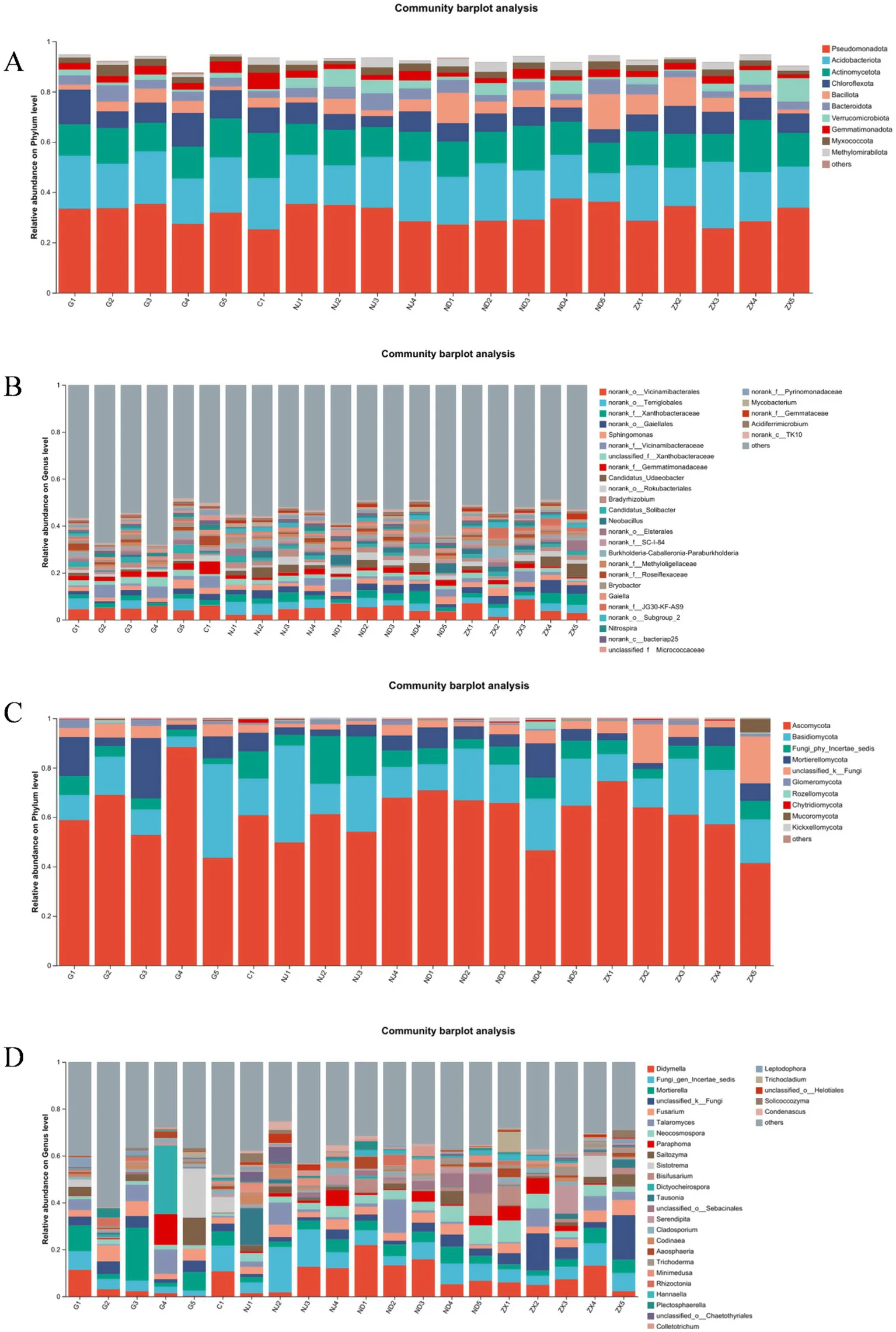
Structural analysis of rhizosphere soil microbial communities at phylum and genus levels. **(A)** Bacterial community at phylum level in rhizosphere soil; **(B)** Bacterial community at genus level in rhizosphere soil; **(C)** Fungal community at phylum level in rhizosphere soil; **(D)** Fungal community at genus level in rhizosphere soil.

At the phylum level, the top three most abundant phyla in ND5 were Pseudomonadota, Bacillota, and Actinomycetota, while in other samples, the top three were Pseudomonadota, Acidobacteriota, and Actinomycetota, with a combined relative abundance exceeding 50%, indicating these as dominant phyla. Although the dominant phyla were similar across regions, differences in relative abundance were observed. For example, Bacillota abundance was relatively higher in ND1, ND5, and ZX2 than in other groups, while Gemmatimonadota abundance was relatively higher in C1. Verrucomicrobiota showed a higher abundance trend in several Anhui samples (NJ1, NJ2, NJ3, ND2, ND4, ZX4, ZX5).

At the genus level, taxa such as *norank_o__Vicinamibacterales*, *norank_o__Terriglobales*, *norank_f__Xanthobacteraceae*, *norank_o__Gaiellales*, and *Sphingomonas* were widely distributed across most samples and constituted the core bacterial community of the Qianhu rhizosphere. However, their relative abundance and dominance varied among regions. In geo-authentic samples (NJ, ND, ZX), *norank_o__Vicinamibacterales* showed high abundance and ranked first in several samples (e.g., ZX3, ZX1, ND1, ND3), representing a key dominant group. *norank_f__Xanthobacteraceae* exhibited local dominance in ND4, ZX5, ZX4, and NJ3. *norank_o__Gaiellales* was consistently present across geo-authentic samples, with relatively high abundance in ZX4, NJ2, and ND3. *Candidatus_Udaeobacter* showed localized enrichment in ZX5, NJ2, ZX4, and ND4. *Sphingomonas* was detected in nearly all geo-authentic samples, with an abundance of approximately 0.02, indicating its role as a common functional genus in the rhizosphere. In other regions, *norank_o__Vicinamibacterales* was also dominant in Guizhou samples, although *norank_o__Terriglobales* was absent in some samples (e.g., G2 and G4). In Chongqing samples, *norank_o__Vicinamibacterales* and *norank_f__Gemmatimonadaceae* were dominant, showing a community structure similar to that of Guizhou.

The composition of rhizosphere fungal communities at the phylum and genus levels is shown in Figures 5C,D.

At the phylum level, the top five phyla in NJ2 were Ascomycota, Fungi_phy_Incertae_sedis, Basidiomycota, Mortierellomycota, and Glomeromycota. In other groups, the dominant phyla were Ascomycota, Basidiomycota, Fungi_phy_Incertae_sedis, Mortierellomycota, and unclassified_k__Fungi. Among these, Ascomycota had the highest relative abundance (41.36–98.23%). Although dominant phyla were similar across regions, their abundances differed. For example, Ascomycota was more abundant in G4, Fungi_phy_Incertae_sedis was higher in NJ2 and NJ3, unclassified_k__Fungi was higher in ZX2 and ZX5, and Basidiomycota was higher in G5. Glomeromycota showed a higher proportion in NJ2 compared with other samples.

At the genus level, *Didymella*, Fungi_gen_Incertae_sedis, *Mortierella*, *Neocosmospora*, *Talaromyces*, and *Fusarium* were widely distributed and constituted the core fungal community. However, their dominance and abundance varied among regions. *Didymella* showed high abundance in most geo-authentic samples, ranking first or second in ND1, ND3, NJ3, NJ4, and ZX4, indicating its role as a key dominant genus. *Neocosmospora* was widely distributed across geo-authentic samples and showed relatively high abundance in ND5, ZX1, and ND4. Fungi_gen_Incertae_sedis was commonly present with high abundance in NJ2, NJ3, and ZX4. *Talaromyces* showed local dominance in ND2, NJ2, and ZX2. In other regions, *Fusarium* and unclassified_k__Fungi were dominant in G2, while *Fusarium* showed particularly high abundance in G3. In G4, *Paraphoma* and *Talaromyces* were dominant, whereas *Saitozyma* was dominant in G5. Guizhou samples exhibited high variability without a consistent community structure, likely due to local environmental differences. In the Chongqing samples, Fungi_gen_Incertae_sedis and *Mortierella* were dominant, with a structure similar to that of the Guizhou samples.

#### 3.2.5 Differential analysis of rhizosphere microbial species

Based on intergroup difference testing, microbial community abundance data were used to test for significant differences in species abundance among rhizosphere soil samples from different origins.

For bacterial communities, multiple-group comparison analysis (Figure 6A) identified significant differences in taxa including *norank_o__Vicinamibacterales*, *norank_o__Terriglobales*, *norank_f__Xanthobacteraceae*, *norank_o__Gaiellales*, *Sphingomonas*, *norank_f__Vicinamibacteraceae*, *norank_f__Gemmatimonadaceae*, *Candidatus_Udaeobacter*, *norank_o__Rokubacteriales*, and *Bradyrhizobium.*

**Figure 6:**
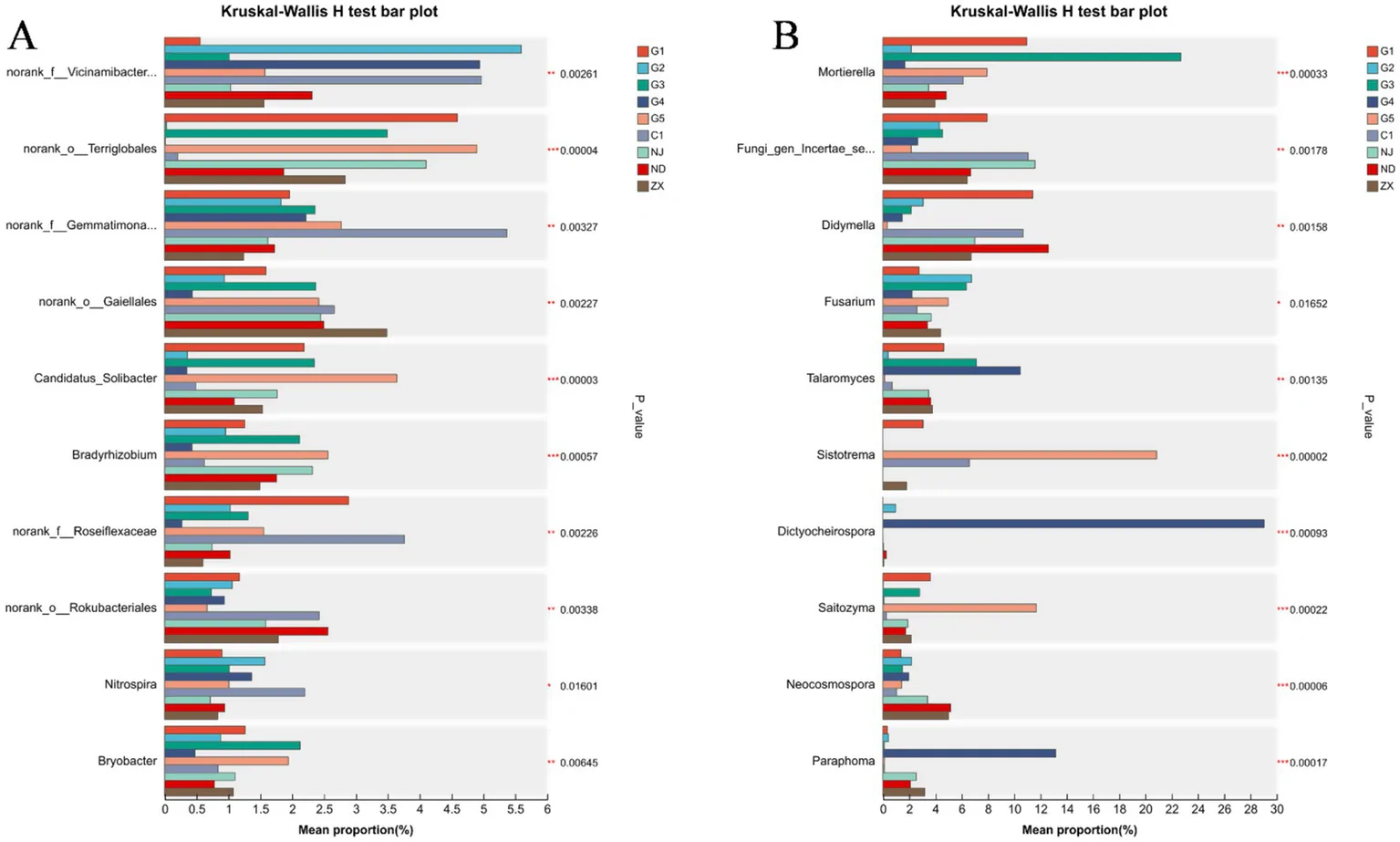
Comparative analysis of rhizosphere soil microbial communities across multiple sample groups. **(A)** Bacteria; **(B)** Fungi.

By combining genus-level community structure analysis and differential analysis, the top 10 most abundant genera and the significantly different taxa were identified as key bacteria. These included *norank_o__Vicinamibacterales*, *norank_o__Terriglobales*, *norank_f__Xanthobacteraceae*, *norank_o__Gaiellales*, *Sphingomonas*, *norank_f__Vicinamibacteraceae*, *norank_f__Gemmatimonadaceae*, *Candidatus_Udaeobacter*, *norank_o__Rokubacteriales*, *Bradyrhizobium*, *Candidatus_Solibacter*, *norank_f__Roseiflexaceae*, *Bryobacter*, and *Nitrospira* (14 taxa in total).

For fungal communities, multiple-group comparison analysis (Figure 6B) identified significant differences in taxa, including *Mortierella*, Fungi_gen_Incertae_sedis, *Didymella*, *Fusarium*, *Talaromyces*, *Sistotrema*, *Dictyocheirospora*, *Saitozyma*, *Neocosmospora*, and *Paraphoma*.

Combining genus-level structure analysis and differential analysis, key fungal taxa were identified, including *Didymella*, Fungi_gen_Incertae_sedis, *Mortierella*, *Fusarium*, *Talaromyces*, *Neocosmospora*, *Paraphoma*, *Saitozyma*, *Sistotrema*, *Bisifusarium*, and *Dictyocheirospora* (11 taxa in total).

### 3.3 Contents of major active components in Qianhu from different origins

The contents of multiple marker components in Qianhu samples are shown in Table 6. According to the requirements of the Chinese Pharmacopoeia (2025 edition, Volume I), the content of PA in Peucedani Radix should not be less than 0.90%, and the content of PB should not be less than 0.24% (calculated on the dried basis). Based on these criteria, among the 20 batches of Qianhu samples, the contents of PA and PB in the Chongqing sample (C1) met the pharmacopoeial standards. In the Guizhou samples (G1, G2, G3, G4, and G5), the PA content met the pharmacopoeial standard, with G2 showing the highest at 1.76%. Among the Ningguo samples, the PA contents of ND1, ND3, ND5, ZX3, ZX4, and ZX5 met the pharmacopoeial standard. The PB content of ND4 also met the standard and was the highest among all samples, reaching 0.25%.

**Table 6:** The content of indicator components in Peucedani Radix from different producing regions (*x* ± *s*, *n* = 3).

Group	Praeruptorin A (%)	Praeruptorin B (%)
C1	0.99 ± 0.05	0.16 ± 0.01
G1	1.16 ± 0.01	0.04 ± 0.00
G2	1.76 ± 0.01	0.07 ± 0.00
G3	1.29 ± 0.00	0.03 ± 0.01
G4	1.27 ± 0.34	0.05 ± 0.01
G5	1.17 ± 0.03	0.04 ± 0.00
NJ1	0.78 ± 0.00	0.11 ± 0.00
NJ2	0.78 ± 0.13	0.07 ± 0.01
NJ3	0.57 ± 0.04	0.16 ± 0.01
NJ4	0.48 ± 0.01	0.23 ± 0.00
ND1	1.31 ± 0.05	0.04 ± 0.00
ND2	0.7 ± 0.02	0.21 ± 0.00
ND3	1.08 ± 0.07	0.08 ± 0.00
ND4	0.61 ± 0.01	0.25 ± 0.00
ND5	1.07 ± 0.01	0.14 ± 0.00
ZX1	0.51 ± 0.08	0.15 ± 0.02
ZX2	0.7 ± 0.00	0.09 ± 0.00
ZX3	1.26 ± 0.05	0.07 ± 0.00
ZX4	1.48 ± 0.11	0.02 ± 0.01
ZX5	1.17 ± 0.01	0.05 ± 0.00

### 3.4 Correlation analysis of aroma, microbial diversity and quality

#### 3.4.1 Correlation analysis between key microbial taxa and aroma components

A total of 28 aroma components were defined as key aroma components, including 10 compounds with ROAV > 1: 2-methylbutanoic acid, methyl ester; allyl sulfide-D; allyl sulfide-M; heptaldehyde-M; heptaldehyde-D; 2,3-pentadione; 1-butanol, 3-methyl-M; alpha-pinene; 1-butanol, 3-methyl-D; and acetic acid-M. In addition, 18 aroma components with relatively higher contents in geo-authentic Ning Qianhu were included: 43, acetaldehyde, acrylonitrile, 51, 38, 2-butanone, 3-hydroxy-M, 6, 2-butanone, 3-hydroxy-D, propanol-D, 31, 4-methyl-2-pentanone-M, 22, 13, (E)-2-hexenal-D, (E)-2-hexenal-M, 1-pentanol-D, 1-hexanol-M, and 1-hexanol-D.

To explore the microbial ecological factors associated with characteristic aroma formation in Qianhu from different origins, Spearman correlation analysis was performed between key bacterial genera and major aroma compounds, and a heatmap was generated (Figure 7).

**Figure 7:**
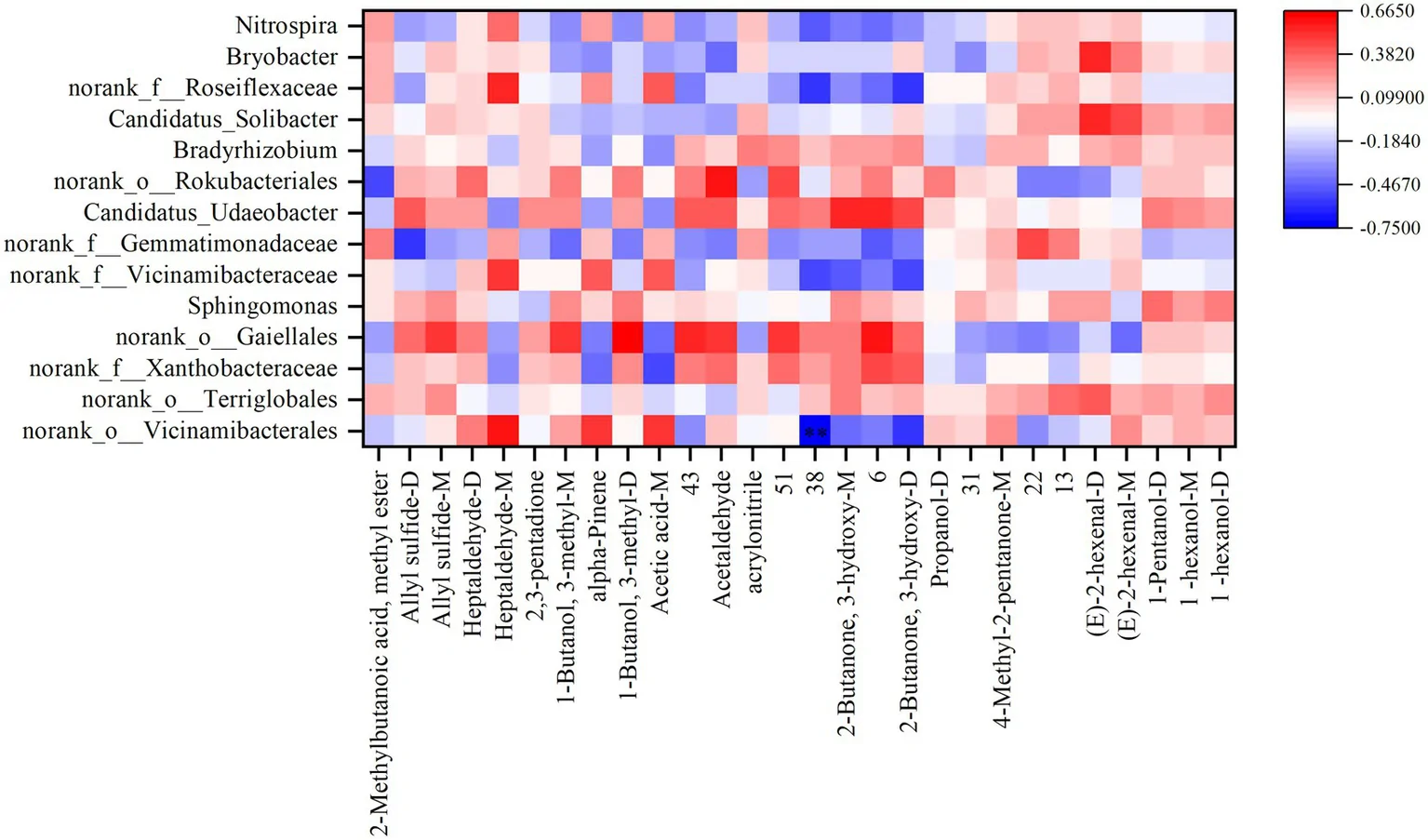
Heatmap of correlation analysis between key bacteria and main flavor compounds. Asterisks indicate the level of statistical significance after FDR correction (** adjusted *p* < 0.01).

After FDR correction, only compound 38 showed a significant negative correlation with *norank_o__Vicinamibacterales* (adjusted *p* < 0.05). Other correlations did not reach statistical significance at the adjusted *p* < 0.05 level, although some genera such as *norank_o__Gaiellales*, *norank_f__Vicinamibacteraceae*, *Candidatus_Udaeobacter*, and *Candidatus_Solibacter* showed consistent association trends with specific volatile compounds prior to correction. These trends, while not statistically robust after correction, may still provide preliminary clues for future targeted investigations. Therefore, we have interpreted these results with caution, emphasizing the exploratory nature of the correlation analysis and the need for independent validation in future studies.

To further clarify the contribution of microbial communities to characteristic aroma formation in Qianhu, Spearman correlation analysis was performed between key fungal genera and major aroma compounds, and a heatmap was generated (Figure 8).

**Figure 8:**
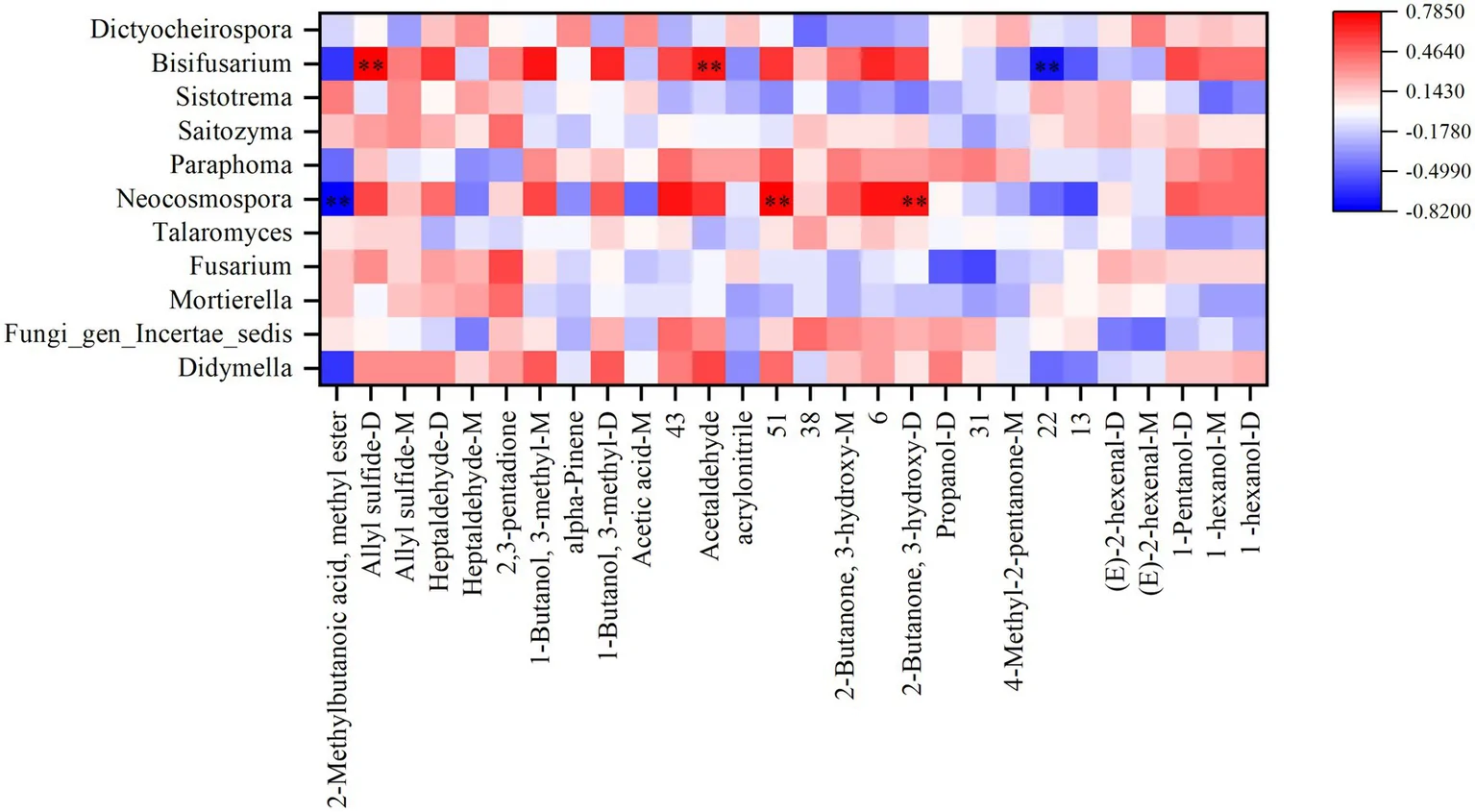
Heatmap of correlation analysis between key fungi and main flavor compounds. Asterisks indicate the level of statistical significance after FDR correction (** adjusted *p* < 0.01).

*Neocosmospora* was extremely significantly positively correlated with compound 51 and 2-butanone, 3-hydroxy-D. In contrast, *Neocosmospora* was extremely significantly negatively correlated with 2-methylbutanoic acid, methyl ester. *Bisifusarium* was extremely significantly positively correlated with allyl sulfide-D and acetaldehyde, but extremely significantly negatively correlated with compound 22.

#### 3.4.2 Correlation analysis between key microbial taxa and major active components

To investigate the potential influence of soil microbial communities on the accumulation of active components in Qianhu, Spearman correlation analysis was performed between key bacterial genera and the contents of PA and PB. The results are shown in Figure 9A.

**Figure 9:**
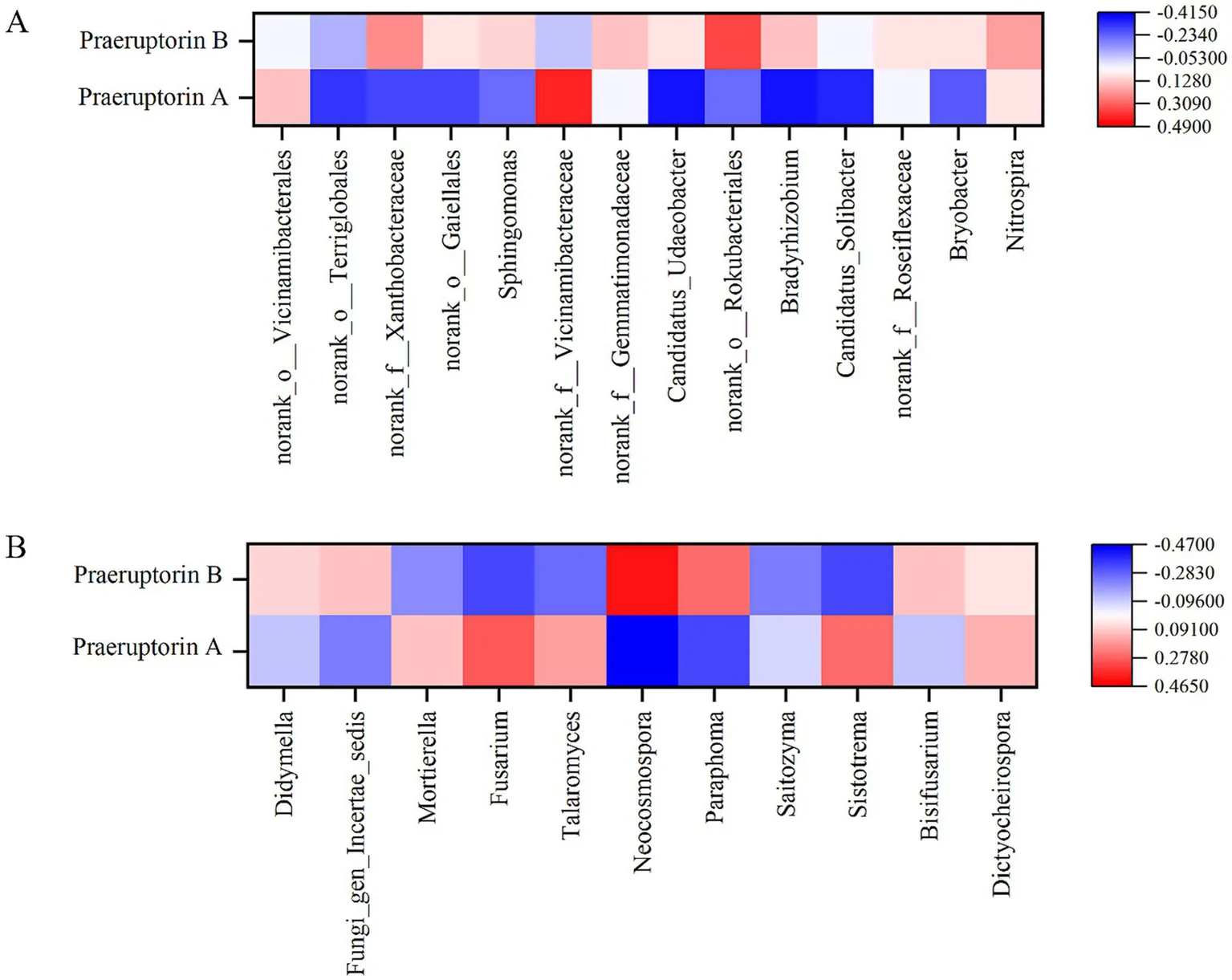
Heatmap of correlation analysis between key microbial genera and major active components. **(A)** Bacteria; **(B)** Fungi.

Before FDR correction, PA showed a positive correlation trend with *norank_f__Vicinamibacteraceae*, and PB showed a positive correlation trend with *norank_o__Rokubacteriales*. However, after FDR correction, no statistically significant correlations were observed between key bacterial genera and PA or PB contents at the adjusted significance level. These trends, while not statistically robust after correction, may still provide preliminary clues for future targeted investigations.

To further investigate the potential influence of soil fungal communities on active component accumulation, Spearman correlation analysis was performed between key fungal genera and PA and PB contents, as shown in Figure 9B.

Before FDR correction, *Neocosmospora* showed a negative correlation trend with PA and a positive correlation trend with PB. However, after FDR correction, no significant correlations were found between key fungal genera and PA or PB contents at the adjusted significance level. Therefore, the correlation results should be interpreted with caution, emphasizing the exploratory nature of the analysis and the need for further validation with larger sample sizes in future studies.

#### 3.4.3 Correlation analysis between major aroma components and major active components

To explore the potential relationship between characteristic aroma compounds and active components in Qianhu, Spearman correlation analysis was conducted between major aroma components and PA and PB contents (Figure 10).

**Figure 10:**
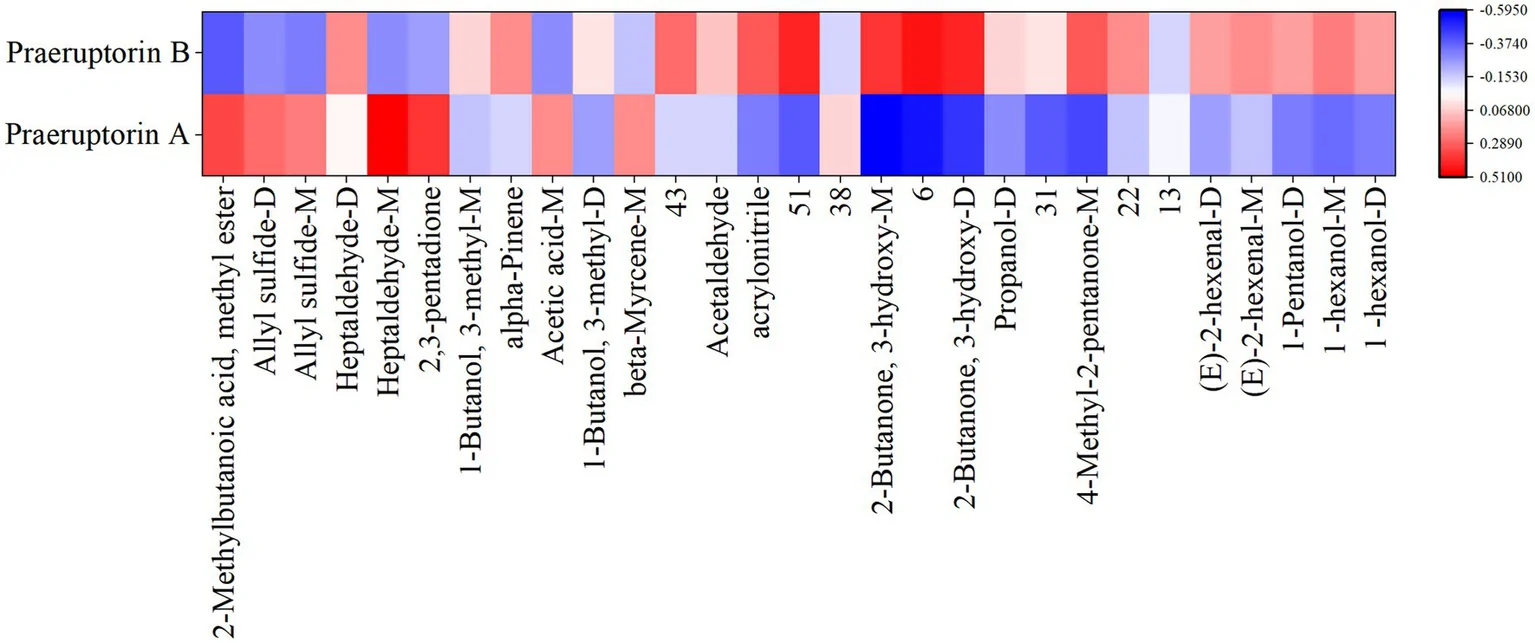
Heatmap of correlation analysis between key microbial genera and main flavor compounds.

Before FDR correction, heptaldehyde-M showed a positive correlation trend with PA, while 2-butanone, 3-hydroxy-M, compound 6, 2-butanone, 3-hydroxy-D, and 4-methyl-2-pentanone-M showed negative correlation trends with PA. For PB, unidentified compound 6 showed a positive correlation trend, while compound 51, 2-butanone, 3-hydroxy-D, 2-butanone, 3-hydroxy-M, and acrylonitrile also showed moderate positive correlation trends. However, after FDR correction, no statistically significant correlations were observed between aroma compounds and PA or PB contents at the adjusted significance level.

These findings suggest that while certain volatile compounds may show association trends with PA and PB accumulation, the current dataset does not provide statistically robust evidence for these relationships after correction for multiple comparisons. The observed trends, though not significant after FDR adjustment, may still offer preliminary clues for future investigations with larger sample sizes.

## 4 Discussion

### 4.1 Aroma characteristics and quality advantages of Qianhu from geo-authentic regions

This study demonstrated that the relative odor activity values (ROAV > 1) of ten key aroma components in Ningguo Qianhu were higher than those in samples from Guizhou and Chongqing. This finding is highly consistent with the traditional quality attribute of Ning Qianhu, characterized by a “strong aroma” (Li and Zhang, 2014; Yang et al., 2012). The application of GC-IMS enabled a systematic characterization of its aroma profile, providing scientific evidence to support traditional sensory-based quality evaluation.

Notably, the volatile oil of Qianhu contains aldehydes such as heptaldehyde and terpenes such as α-pinene. Sensory evaluation studies have shown that the aroma profile of Qianhu volatile oil is mainly characterized by grassy, woody, spicy, and earthy notes, with a pine-resinous nuance, which is highly correlated with terpene components such as β-pinene. These volatile compounds may collectively contribute to the distinctive aroma profile of Qianhu, consistent with the traditional quality evaluation criterion of “strong aroma” in Qianhu.

From a mechanistic perspective, the formation of these volatile compounds is closely linked to plant secondary metabolism. A recent chromosome-scale genome study of P. praeruptorum revealed that the terpene synthase (TPS) gene family is significantly expanded in this species, which may contribute to the abundance of terpenoids in P. praeruptorum (Song et al., 2024). Furthermore, changes in rhizosphere microbial community structure can significantly affect secondary metabolite accumulation in medicinal plants (Zhang et al., 2025). Therefore, the higher ROAV values observed in Ning Qianhu may reflect the combined effects of genetic background and regionally distinct rhizosphere microbial communities on volatile biosynthesis.

### 4.2 Relationship between rhizosphere microbial community structure and geo-authenticity

High-throughput sequencing revealed that the rhizosphere bacterial community in geo-authentic regions was dominated by *norank_o__Vicinamibacterales* and *norank_o__Terriglobales*, while *Didymella* and *Neocosmospora* dominated the fungal community. These taxa showed consistently higher abundances compared with those in non-geo-authentic regions.

Previous studies by Korenblum et al. (2022) reported that fungal taxa such as *Didymella* and *Fusarium* were highly abundant in the rhizosphere of Qianhu during the non-bolting stage. A *Didymella* strain isolated from Qianhu roots was found to contain PA, PB, and praeruptorin E, and field inoculation experiments further confirmed its influence on active component accumulation. These findings support the positive correlations observed between *Didymella* and aroma compounds (e.g., acetaldehyde and isoamyl alcohol), suggesting that *Didymella* may simultaneously promote aroma formation and the accumulation of active components.

### 4.3 Potential mechanisms linking microorganisms, aroma components, and active components

Correlation analysis revealed a complex association network linking rhizosphere microorganisms, aroma compounds, and active components. Within the bacterial community, *Gaiellales* showed the broadest range of positive association trends with aroma compounds, particularly alcohols, aldehydes, and thioethers, suggesting a potentially important role in aroma formation. Members of *Acidobacteriota* (e.g., *Vicinamibacterales*, *Vicinamibacteraceae*, and *Candidatus_Solibacter*) were primarily positively associated with aldehydes and negatively associated with ketones, suggesting their possible involvement in aldehyde biosynthesis or ketone degradation.

Within the fungal community, *Didymella*, *Neocosmospora*, and *Bisifusarium* exhibited positive association trends with alcohol- and aldehyde-type aroma compounds, but negative trends with 2-methylbutanoic acid, methyl ester, indicating similar functional tendencies in flavor formation. Among them, *Neocosmospora* and *Bisifusarium* showed broader associations, including trends with thioethers (e.g., allyl sulfide-D), ketones (e.g., 2-butanone, 3-hydroxy-), and several unidentified compounds (6, 43, 51), suggesting that they may play important roles within the aroma-associated microbial network.

Interestingly, *Neocosmospora* showed opposite association trends with PA and PB, being negatively associated with PA but positively associated with PB, suggesting potential differential roles in coumarin accumulation. This observation is consistent with recent evidence that specific microorganisms can modulate secondary metabolism in Qianhu. For example, *Penicillium restrictum* has been shown to significantly enhance coumarin accumulation and alter related metabolic pathways during co-cultivation (Song et al., 2025). These results provide preliminary mechanistic insights into the microorganism–active component associations observed in the present study.

Furthermore, PA and PB displayed distinct microbial association patterns: PA showed positive association trends with *Vicinamibacteraceae*, whereas PB showed positive association trends with *Rokubacteriales*. These results suggest that different microbial functional groups may be associated with the accumulation of these two coumarins. Metabolomic studies have indicated that phenylpropanoid biosynthesis plays a key role in Qianhu quality formation (Guo et al., 2024), suggesting that microorganisms may differentially influence PA and PB synthesis by modulating this pathway.

### 4.4 Study limitations

It should be noted that bulk soil samples were collected during the sampling process but were not subjected to high-throughput sequencing analysis in the present study. As a result, we are unable to fully distinguish rhizosphere-specific microbial taxa from the background soil community. In addition, the sample size for the Chongqing group was limited to only one biological replicate, which precluded the application of supervised multivariate methods such as OPLS-DA due to the risk of overfitting. Furthermore, the PCA model explained only 44.5% of the total variance, indicating limited discriminatory power. Therefore, the results for Chongqing should be interpreted with caution and considered preliminary. Future studies with larger and more balanced sample sizes, incorporating comparative analysis of bulk soil and rhizosphere soil microbiomes, are needed to further validate and extend our findings using more robust classification models.

A number of unknown volatile compounds (labeled with numbers) were included in the correlation analysis. These compounds were retained because they exhibited relatively high ROAV values and consistent chromatographic behavior across replicated samples, suggesting potential contributions to the aroma profile of Qianhu. However, due to the limited coverage of the current GC-IMS spectral library, these compounds could not be definitively identified. The correlation results involving these unknown compounds should therefore be interpreted with caution, and future studies using GC–MS or reference standards are needed for their structural identification.

### 4.5 Future perspectives

This study provides evidence for the correlations among rhizosphere microorganisms, aroma components, and active constituents in Qianhu, offering new insights into the microecological mechanisms underlying quality formation. Traditional evaluation criteria emphasize “strong aroma” as a key indicator of high-quality Qianhu, highlighting the importance of aroma-related traits. Future studies should focus on isolating and developing functional microbial strains positively associated with aroma formation, such as *Gaiellales*, *Neocosmospora*, and *Bisifusarium*. In addition, the synergistic interactions between dominant microbial taxa and aroma-producing microorganisms warrant further investigation. These efforts may facilitate the development of rhizosphere microbial inoculants, thereby improving geo-authentic quality and promoting standardized cultivation of Qianhu.

## 5 Conclusion

This study systematically analyzed the differences and intrinsic correlations among aroma components, rhizosphere microbial communities, and active constituents (PA and PB) in Qianhu from the geo-authentic region (Ningguo) and other production regions. The results showed that Ningguo Qianhu exhibited advantages in aroma intensity, with 10 key aroma compounds (ROAV > 1) showing higher relative odor activity values than those from other regions. In terms of rhizosphere microbial communities, the dominant bacterial taxa in the geo-authentic region were *norank_o__Vicinamibacterales* and *norank_o__Terriglobales*, while the dominant fungal taxa were Didymella and Neocosmospora. Correlation analysis revealed that different microbial taxa may play differential roles in aroma formation and active constituent accumulation. Among them, *Gaiellales*, *Neocosmospora*, and *Bisifusarium* showed broad associations with multiple aroma components, while PA and PB may be associated with distinct microbial functional groups. However, after FDR correction, none of the correlations between key microbial genera/aroma components and active constituents reached statistical significance; therefore, the findings of this study should be considered exploratory observations. In conclusion, Qianhu from the geo-authentic region showed certain advantageous trends in aroma quality and microbial community structure, but the underlying microecological mechanisms require further validation through expanded sample sizes and experimental approaches. This study provides preliminary exploratory insights and a scientific basis for understanding the microecological mechanisms underlying the geo-authenticity formation of Qianhu.

## Data Availability

SRA data are available in NCBI (www.ncbi.nlm.nih.gov) under the accession number PRJNA1466971.

## Footnotes

1
https://cloud.majorbio.com
